# Supplementary material for: Cas9 targeted enrichment of mobile elements using nanopore sequencing
Source: Nat Commun. 2021 Jun 11;12:3586. doi: 10.1038/s41467-021-23918-y (PMC8196195; doi:10.1038/s41467-021-23918-y)
Supplement: Supplementary file 1 — Supplementary Information [file 41467_2021_23918_MOESM1_ESM.pdf]

Supplementary Information

Cas9 targeted enrichment of mobile elements using nanopore sequencing

Torrin L. McDonald*[,1], Weichen Zhou*[,2], Christopher Castro[2], Camille Mumm[1], Jessica A. Switzenberg[2], Ryan E. Mills[1,2,†], Alan P. Boyle[1,2,†]

[1]Department of Human Genetics, University of Michigan, Ann Arbor, MI, USA

[2]Department of Computational Medicine and Bioinformatics, University of Michigan, Ann Arbor, MI, USA

*****These authors contributed equally

[†]To whom correspondence should be addressed. Email: remills@umich.edu (R.E.M.) and apboyle@umich.edu (A.P.B.)

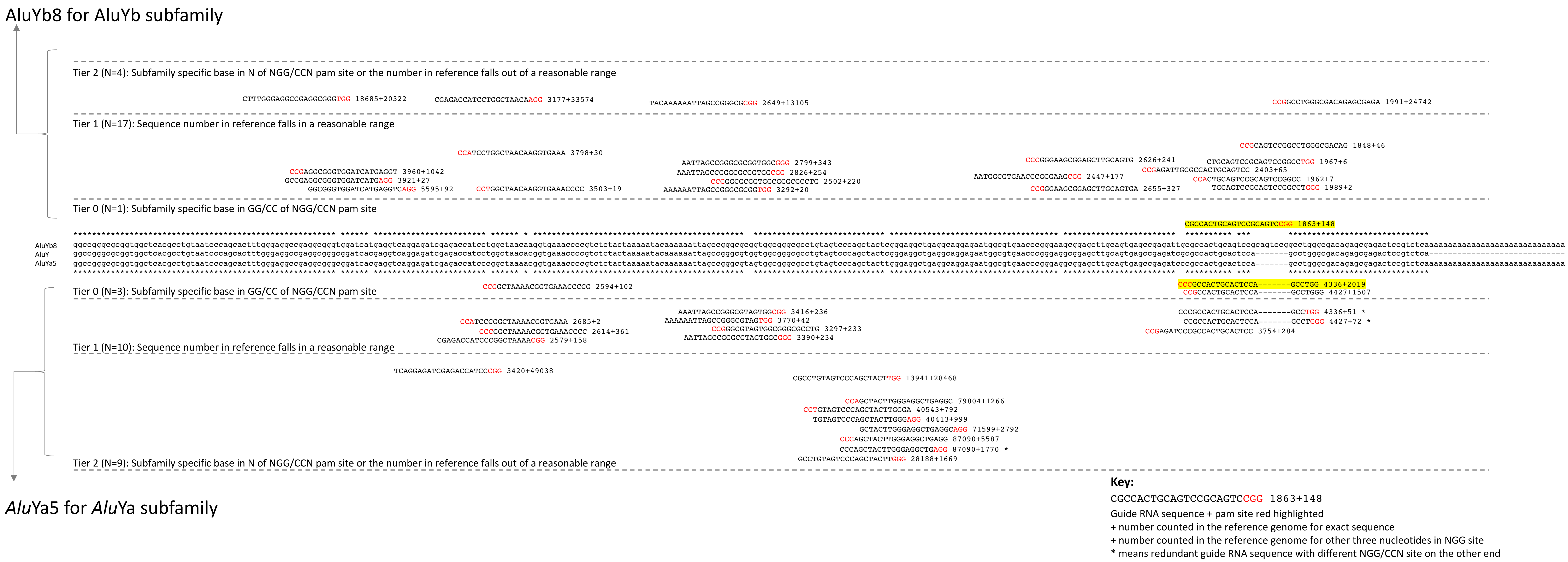

Supplementary Fig.1: Guide RNA design for *Alu*Yb and *Alu*Ya element.
Consensus sequences are aligned and showed for *Alu*Yb, *Alu*Ya, and *Alu*Y in the middle. The candidate guide RNAs are distributed based on different tiers (see **Methods**). The final list is highlighted by yellow.

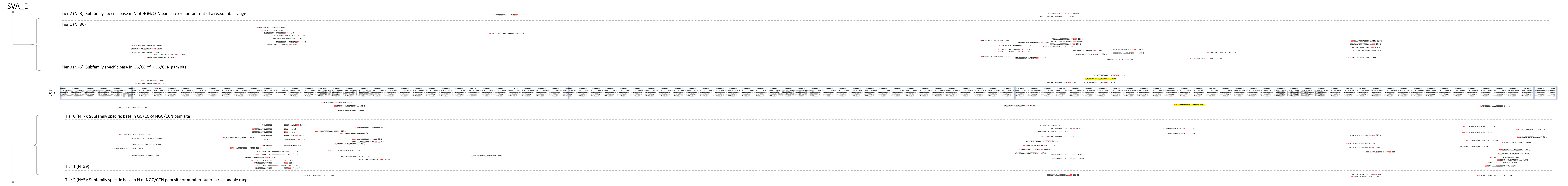

Key:

CGCCACTGCAGTCCGCAGTCCGG 1863+148

Guide RNA sequence + pam site red highlighted

+ number counted in the reference genome for exact sequence

+ number counted in the reference genome for other three nucleotides in NGG site

* means redundant guide RNA sequence with different NGG/CCN site on the other end

Supplementary Fig.2: Guide RNA design for SVA_F and SVA_E element.
Consensus sequences are aligned and showed for SVA_F, SVA_E, and SVA_D in the middle. The candidate guide RNAs are distributed based on different tiers (see **Methods**). The final list is highlighted by yellow.

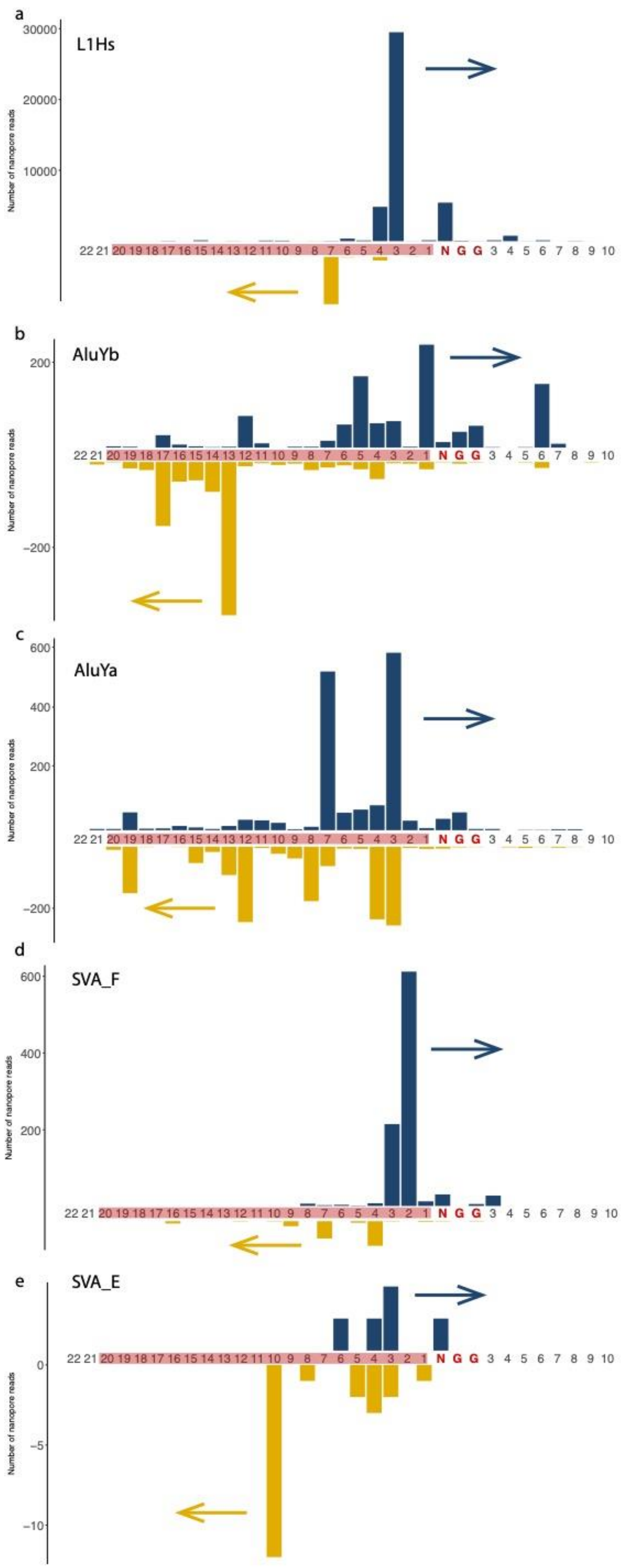
a
L1Hs
Number of nanopore reads
30000
20000
10000
22 21 20 19 18 17 16 15 14 13 12 11 10 9 8 7 6 5 4 3 2 1 N G G 3 4 5 6 7 8 9 10
b
AluYb
200
−200
c
AluYa
600
400
200
−200
d
SVA_F
600
400
200
e
SVA_E
0
−5
−10

Supplementary Fig.3: Distributions of guide RNA cleavage-site for five MEI subfamilies

a, Cleavage-site distribution of L1Hs guide RNA. X-axis shows the position where the read ends or begins with the number indicating the distance from the ‘N’ of the PAM site (NGG). The PAM site (NGG) was colored red and guide RNA bases were highlighted by red background. Y-axis is the number of nanopore reads counted. The upper blue bar represents the reads with forward strand sequencing outward the 3’ end of guide RNA and the lower yellow bar represents the reads with reverse strand sequencing outward the 5’ end of guide RNA. **b,** Cleavage-site distribution of *Alu*Yb guide RNA. **c,** Cleavage-site distribution of *Alu*Ya guide RNA. **d,** Cleavage-site distribution of SVA_F guide RNA. **e,** Cut-site distribution of SVA_E guide RNA.

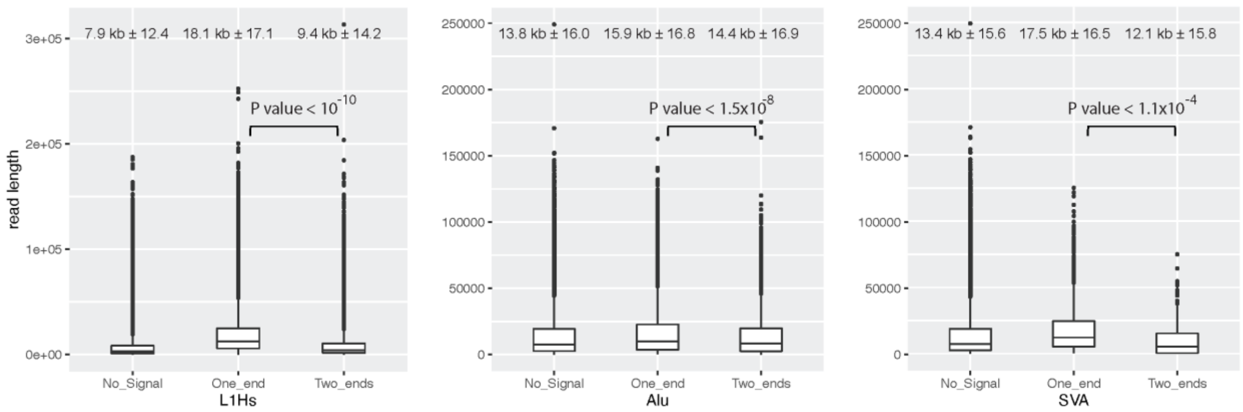

Supplementary Fig.4: Read length distributions for MEI categories (L1Hs, *Alu*Y, and SVA).

All reads were identified into three categories: reads with on-target reference MEI signals, ones with on-target non-reference MEI signals, and ones with no signals (off-target). The read length (mean ± standard deviation) are shown above the boxplots. For L1Hs experiments, n=442,674 reads have no signal, n=244,643 reads have signal on one end, and n=44,059 reads have signals on both ends. For *Alu*Y experiments, n=84,970 reads have no signal, n=30,778 reads have signal on one end, and n=4,460 reads have signals on both ends. For SVA experiments, n=122,585 reads have no signal, n=4,083 reads have signal on one end, and n=148 reads have signals on both ends. And the P-values (student's T-test, two-tailed), $<10^{-10}$, $<1.5\times10^{-8}$, and $<1.1\times10^{-4}$, are shown between one-end reads and two-end reads in the L1Hs, *Alu*Y, and SVA experiments, respectively. Error bars range from Q1-1.5IQR to Q3+1.5IQR (IQR, interquartile range).

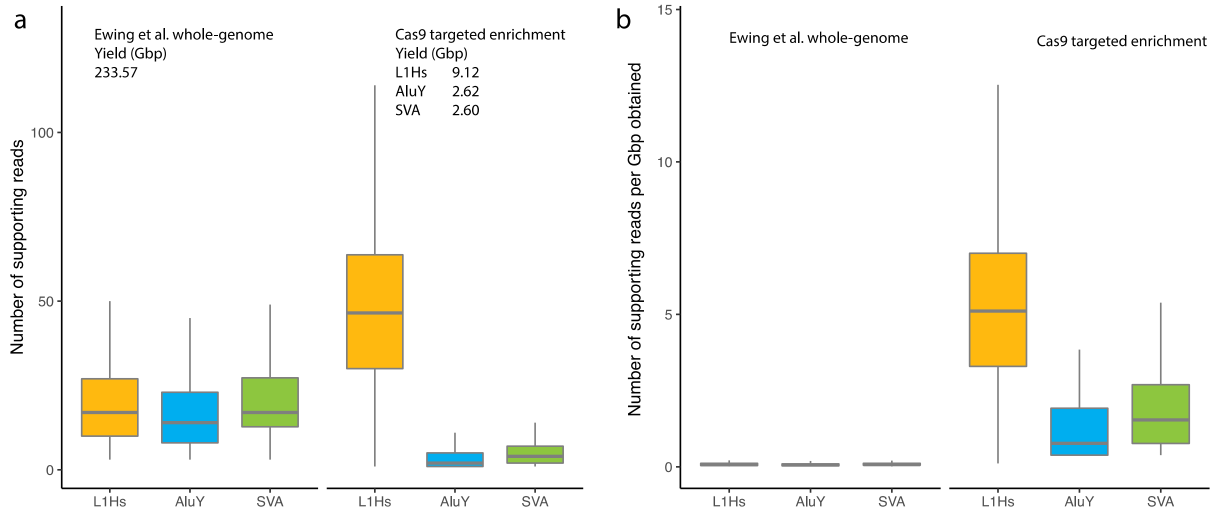

Supplementary Fig.5: Number of supporting reads for three categories of non-reference MEI from the Cas9 targeted nanopore sequencing and the whole-genome nanopore sequencing by Ewing et al. 2020.

a, Number of supporting reads for non-reference L1Hs, *Alu*Y, and SVA in the whole-genome nanopore sequencing (from five PromethION flow cells) and the Cas9 targeted nanopore sequencing (L1Hs from 11 MinION/Flongle flow cells, *Alu*Y from four from 4 MinION/Flongle flow cells, and SVA from 4 MinION/Flongle flow cells). **b**, Number of supporting reads of non-reference MEIs normalized by the total yield base pairs from flow cells in two studies. For whole-genome Ewing et al., there are 20.35 ± 13.26 (mean ± standard deviation) supporting reads and 0.087 ± 0.057 supporting reads per Gbp obtained for non-reference L1Hs (n=311), 17.52 ± 12.01 supporting reads and 0.075 ± 0.051 supporting reads per Gbp obtained for non-reference *Alu*Y (n=2359), and 21.43 ± 11.85 supporting reads and 0.092 ± 0.051 supporting reads per Gbp obtained for non-reference SVA (n=108). For Cas9 targeted enrichment experiments, there are 49.24 ± 31.25 supporting reads and 5.41 ± 3.43 supporting reads per Gbp obtained for non-reference L1Hs (n=194), 3.59 ± 3.29 supporting reads and 1.38 ± 1.26 supporting reads per Gbp obtained for non-reference *Alu*Y (n=606), and 4.88 ± 3.79 supporting reads and 1.88 ± 1.46 supporting reads per Gbp obtained for non-reference SVA (n=49). The error bars of boxplot range from Q1-1.5IQR to Q3+1.5IQR (IQR, interquartile range).

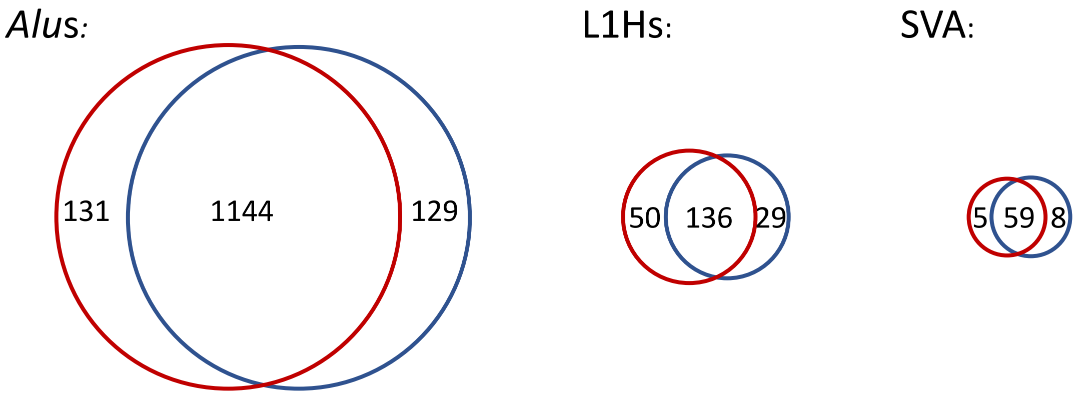

Supplementary Fig.6: Venn diagram of the PALMER callset and the PAV callset for non-reference MEIs in NA12878 genomes.

PALMER callset (red circle) is from PacBio raw sub-reads, and PAV (the Phased Assembly Variant caller, https://github.com/EichlerLab/pav, see **Methods**) callset (blue circle) is from PacBio assembly-based pipeline. The circles are depicted by the scale of the numbers showed inside. The union of two sets is generated as 'PacBio-MEI' to be the gold standard set to compare with the calls from nanopore data.

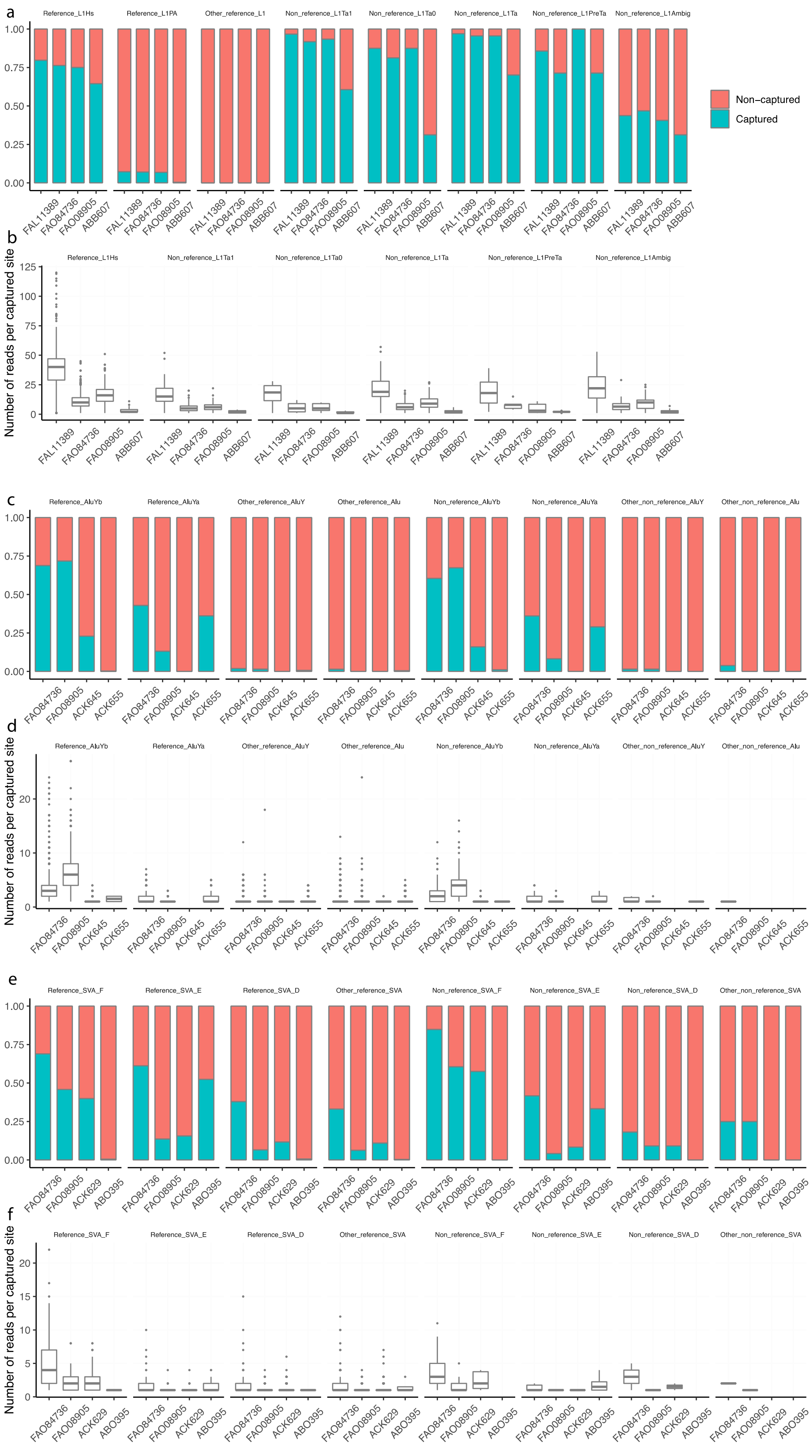
a
Reference_L1Hs
Reference_L1PA
Other_reference_L1
Non_reference_L1Ta1
Non_reference_L1Ta0
Non_reference_L1Ta
Non_reference_L1PreTa
Non_reference_L1Ambig
1.00
0.75
0.50
0.25
0.00
FAL11389
FAO84736
FAO08905
ABB607
Non−captured
Captured
b
Number of reads per captured site
Reference_L1Hs
Non_reference_L1Ta1
Non_reference_L1Ta0
Non_reference_L1Ta
Non_reference_L1PreTa
Non_reference_L1Ambig
125
100
75
50
25
0
c
Reference_AluYb
Reference_AluYa
Other_reference_AluY
Other_reference_Alu
Non_reference_AluYb
Non_reference_AluYa
Other_non_reference_AluY
Other_non_reference_Alu
FAO84736
FAO08905
ACK645
ACK655
d
Number of reads per captured site
20
10
0
e
Reference_SVA_F
Reference_SVA_E
Reference_SVA_D
Other_reference_SVA
Non_reference_SVA_F
Non_reference_SVA_E
Non_reference_SVA_D
Other_non_reference_SVA
FAO84736
FAO08905
ACK629
ABO395
f
Number of reads per captured site
20
15
10
5
0

Supplementary Fig.7: Summary of recovered known reference and non-reference MEIs.

a, Known L1Hs in GM12878 recovered by Cas9 targeted enrichment from the individual MinION flow cell (FAL11389), pooled-MEI MinION flow cell (FAO84736), and individual Flongle flow cell (ABB607), displayed in a way of proportion of the upper-bound known reference L1Hs, L1Pa, and other L1 as well as non-reference (non-ref.) subfamilies (L1Ta1, L1Ta0, L1Ta, L1PreTa, and L1Hs with ambiguous subfamilies) of L1Hs from PacBio-MEI set. **b,** The number of supporting reads in each captured L1 in the context of **a.** **c,** Known *Alu*Y elements in GM12878 recovered by Cas9 enrichment in two pooled MinION flow cells (FAO84736 and FAO08905), one individual *Alu*Yb Flongle flow cell (ACK645), and one individual *Alu*Ya Flongle flow cell (ACK655). **d,** The number of supporting reads in each captured *Alu* element in the context of **c**. **e,** Known SVA elements in GM12878 recovered by Cas9 enrichment in two pooled MinION flow cells (FAO84736 and FAO08905), one individual SVA_F Flongle flow cell (ACK629), and one individual SVA_E Flongle flow cell (ACK395). **f,** The number of supporting reads in each captured *Alu* element in the context of **e**. In **b, d, f**, the numbers of captured MEI subfamily in the boxplots can be found in **Supplementary Data 6** with information of mean and standard deviation; The error bars of boxplot range from Q1-1.5IQR to Q3+1.5IQR (IQR, interquartile range).

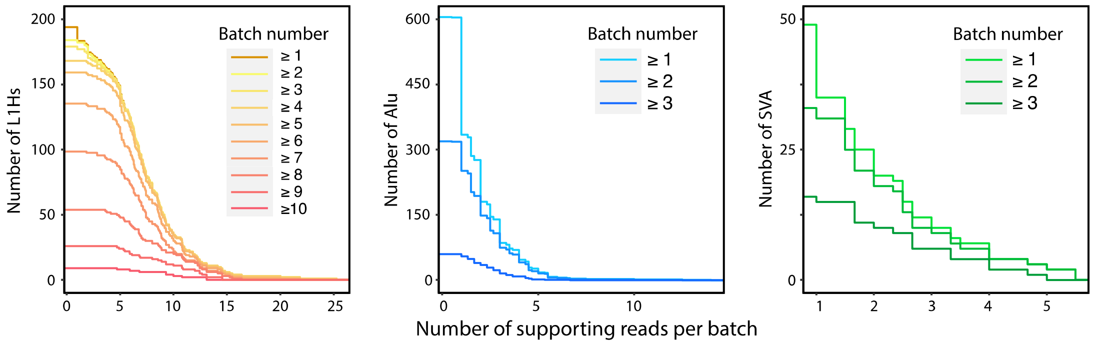

Supplementary Fig.8: MEI distributions in various number of flow cells.

The number of MEIs (L1Hs, yellow; *Alu*Y, blue; SVA, green) can be captured by nanopore Cas9 enrichment regarding different numbers of flow cells and cutoffs of supporting reads.

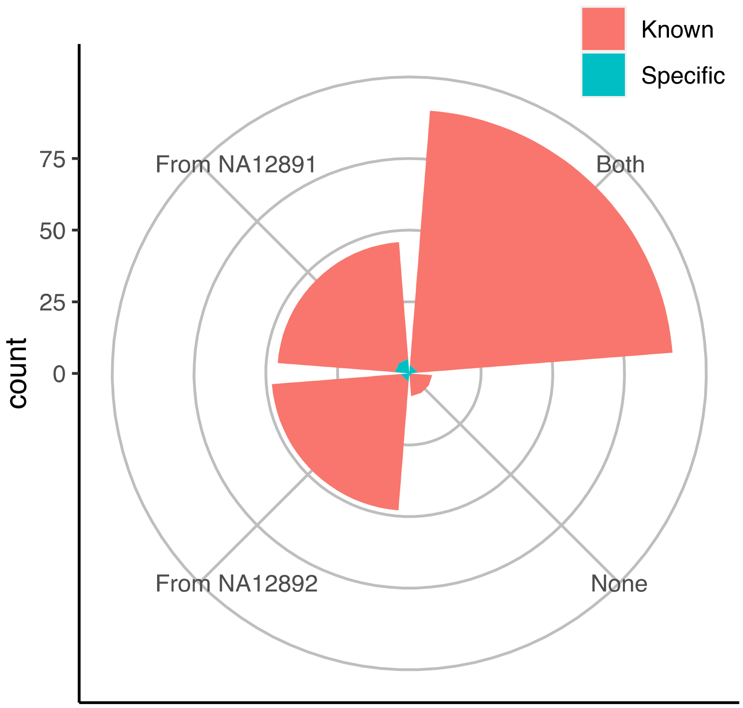

Supplementary Fig.9: Trio transmission of 194 non-reference L1Hs captured by nanopore in GM12878 sample.

The intersections with ‘PacBio-MEI’ were shown by red and the nanopore-specific non-reference L1Hs that were missed by ‘PacBio-MEI’ were shown by green.

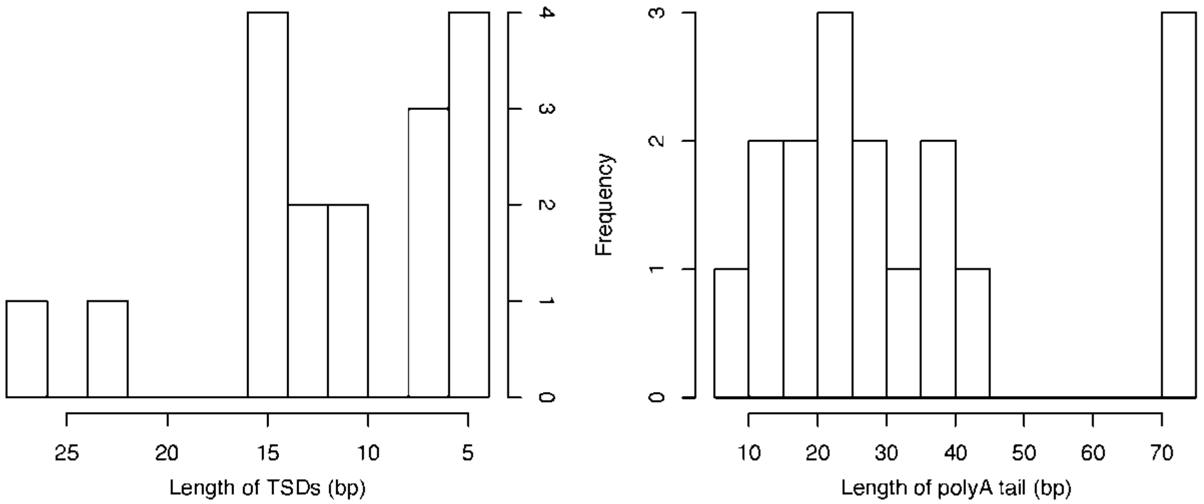

Supplementary Fig.10: Seventeen nanopore-specific MEIs have hallmarks of retrotransposition consistent with bona fide insertions.

Left, length distribution of TSD motifs. Right, length distribution of poly(A) tails.

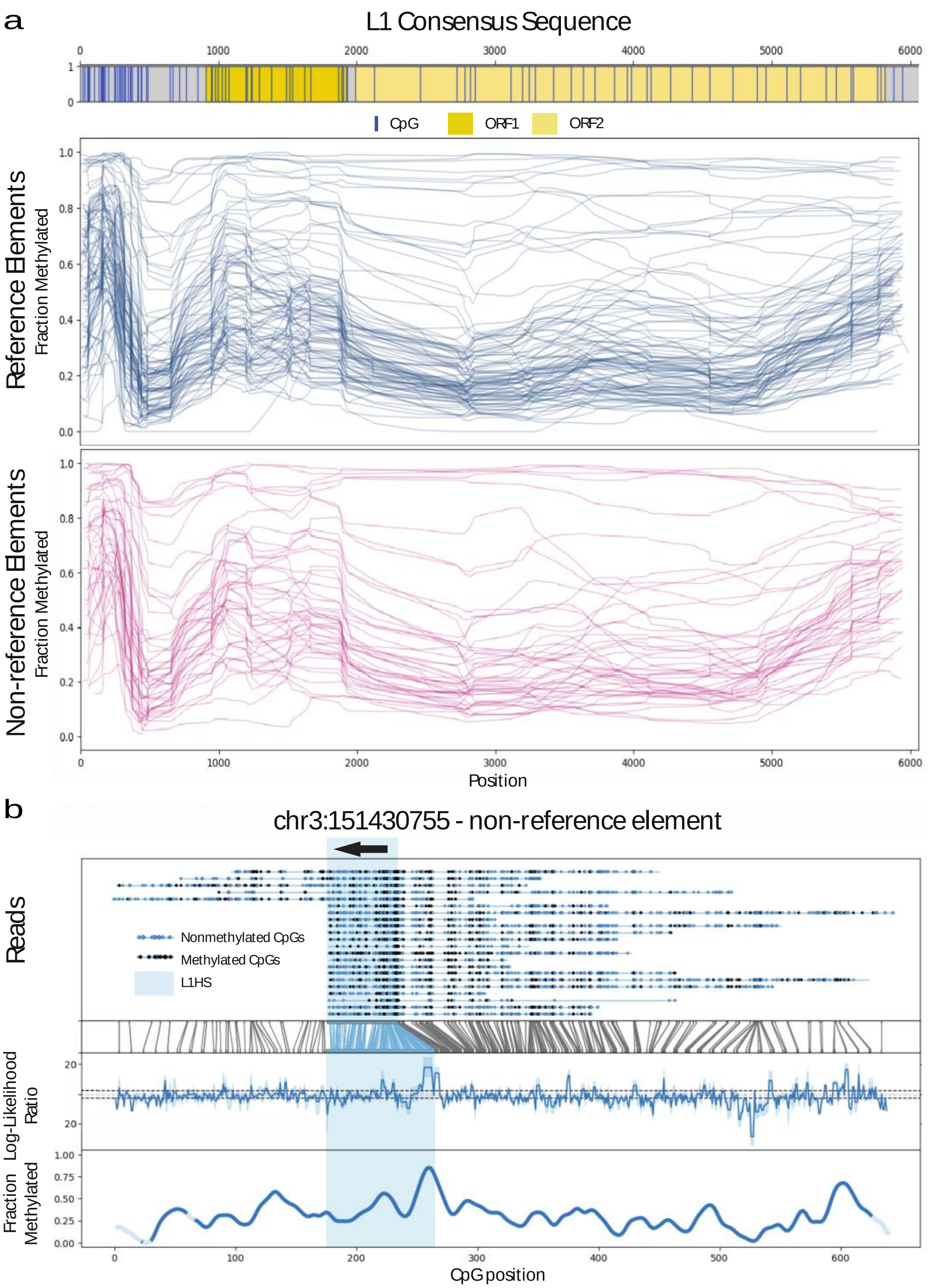

Supplementary Fig.11: Examing CpG methylation of captured L1Hs reads.

a, L1Hs methylation profile over consensus L1 sequence in reference (blue) and non-reference elements (magenta). **b**, An example of methylation profile at chr3:151430755 non-reference L1Hs (black arrow 3'->5' orientation). Individual reads with methylation profiles are shown (top) along with their aggregate methylation profile (bottom).
