# Supplementary material for: Cas9 targeted enrichment of mobile elements using nanopore sequencing
Source: Nat Commun. 2021 Jun 11;12:3586. doi: 10.1038/s41467-021-23918-y (PMC8196195; doi:10.1038/s41467-021-23918-y)
Supplement: Supplementary file 2 — Description of Additional Supplementary Files [file 41467_2021_23918_MOESM2_ESM.pdf]

Title: Supplementary Data 1.

Description: A list of guide RNA candidates

Sheet1, five final guide RNAs in the project for five MEIs, along with the information of the counts of forward/reverse strand reads based on these guide RNA sequence. Sheet2, all candidate guide RNAs for L1Hs that mapping to unique sequences of L1Hs subfamily followed by the frequency in the reference genome sequence. Sheet3 for AluYb, Sheet4 for AluYa, Sheet5 for SVA_F, and Sheet6 for SVA_E.

Title: Supplementary Data 2.

Description: Counts of forward and reverse reads in cleavage-site analysis.

Title: Supplementary Data 3.

Description: Master table of Information for all flow cells in the project

Sheet1, information of reads from all 17 flow cells. Sheet2, information of reads after MEI classification by Nano-Pal. Sheet3, number of MEI events captured by each flow cell after reads clustering.

Title: Supplementary Data 4.

Description: Non-reference MEI callsets from PacBio-MEI and MELT.

Sheet1, PacBio-MEI L1Hs callset in GM12878. Sheet2, PacBio-MEI Alu callset in GM12878. Sheet3, PacBio-MEI SVA callset in GM12878. Sheet4, MELT L1Hs callset for GM12891. Sheet5, MELT L1Hs callset for GM12892.

Title: Supplementary Data 5.

Description: Known MEIs captured by nanopore Cas9 enrichment approach in different flow cells based on different boundaries.

Upper-bound, intermediate, and lower-bound values of different categories of MEIs are included regarding background (number) and seven representative flow cells(percentage).

Title: Supplementary Data 6.

Description: Statistical summary of recovered known reference and non-reference MEIs.

Sheet1, statistical summary of recovered known reference and non-reference MEIs for Fig. 3b,d,f. Sheet2, statistical summary of recovered known reference and non-reference MEIs for Fig. 3h. Sheet3, statistical summary of recovered known reference and non-reference MEIs for Supplementary Fig. 3b,d,f.

Title: Supplementary Data 7.

Description: Enrichment of mobile element signals in nanopore reads from GM12878 trio L1Hs experiments

Four flow cells were carried out for trio experiments in the project: three individual Flongle flow cells for GM12878 (ABG188), GM12891 (ABO515), and GM12892 (ABN780) each, and one MinION flow cell for pooled three samples (FAL15177).

Title: Supplementary Data 8.

Description: Information for nanopore-specific captured non-reference MEIs and examples

Sheet1, summary of potential nanopore-specific non-reference MEIs in different categories (see Methods). Sheet2, information of potential nanopore-specific non-reference L1Hs. Sheet3, information of potential nanopore-specific non-reference AluY elements. Sheet4, information of potential nanopore-specific non-reference SVAs. Sheet5, IGV screenshots for an L1Hs example at chrX:121709062-121709136 as true positive. Sheet6-8, IGV screenshots of examples of L1Hs identified as false positives. Sheet9,10, IGV screenshots for examples of AluYs identified as true positives. Sheet11-13, IGV screenshots for examples of AluYs and SVAs identified as false positives.

Title: Supplementary Data 9.

Description: Information of 17 nanopore-specific MEIs (12 L1Hs and 5 AluY) captured by Cas9 targeted enrichment and missed by PacBio-MEI.

Each event has two sections: general information and IGV screenshot. In the general information section, we show a) length, strand, genotype of the insertion event, b) empty site with sequence information, c) information of hallmarks indicating a retrotransposition, including lengths of TSD, poly(A), and EN Cleavage site information, and d) genotype information in NA12878, sample frequency and allele frequency in 32 genomes reported in Ebert et al. 2021. Science. In the section of IGV screenshot, we included a) true-positive type for this insertion (see Methods and Supplementary Data 7), and b) IGV screenshot in a range of genomic region with seven tracks: Cas9 targeted nanopore reads, assembled PacBio contig haplotype one by Ebert et al. 2021. Science, assembled PacBio contig haplotype two by Ebert et al. 2021. Science, sequence track, gene track, segmental duplication track, RepeatMasker track.

Title: Supplementary Data 10.

Description: MEI callset by Cas9 targeted enrichment using nanopore sequencing in GM12878

Sheet1, information for the L1Hs callset in GM12878. Sheet2, information for the AluY callset in GM12878. Sheet3, information for the SVA callset in GM12878.
