# Supplementary material for: Cas9 targeted enrichment of mobile elements using nanopore sequencing
Source: Nat Commun. 2021 Jun 11;12:3586. doi: 10.1038/s41467-021-23918-y (PMC8196195; doi:10.1038/s41467-021-23918-y)

L1Hs call at chr1: 83144052

✧ General information

L1Hs insertion: 1742bp L1Hs seq (-) 0/1

5' CAGAGCTGCAACAGGCCGAA AGCC **Insertion** AGCC CAGGTTGGGTGTTTCTG 3'

➢ Empty site

5' CAGAGCTGCAACAGGCCGAA AGCC CAGGTTGGGTGTTTCTG 3'
3' GTCTCGACGTTGTCCGGCTT TCGG GTCCAACCCACAAAGAC 5'

➢ Hallmarks

TSD length: 4bp
polyA length: 11bp
EN Cleavage site: 5' AGCC/C 3'

➢ In Ebert et al. 2021. Science

NA12878 genotype:	0/0
Sample frequency:	6/32
Allele frequency:	9/64

✧ IGV screenshot (general information can be defined by nanopore reads)

➢ True positive type2 (as missed by other callers, PALMER and PAV)

chr1:83,144,052-83,144,114

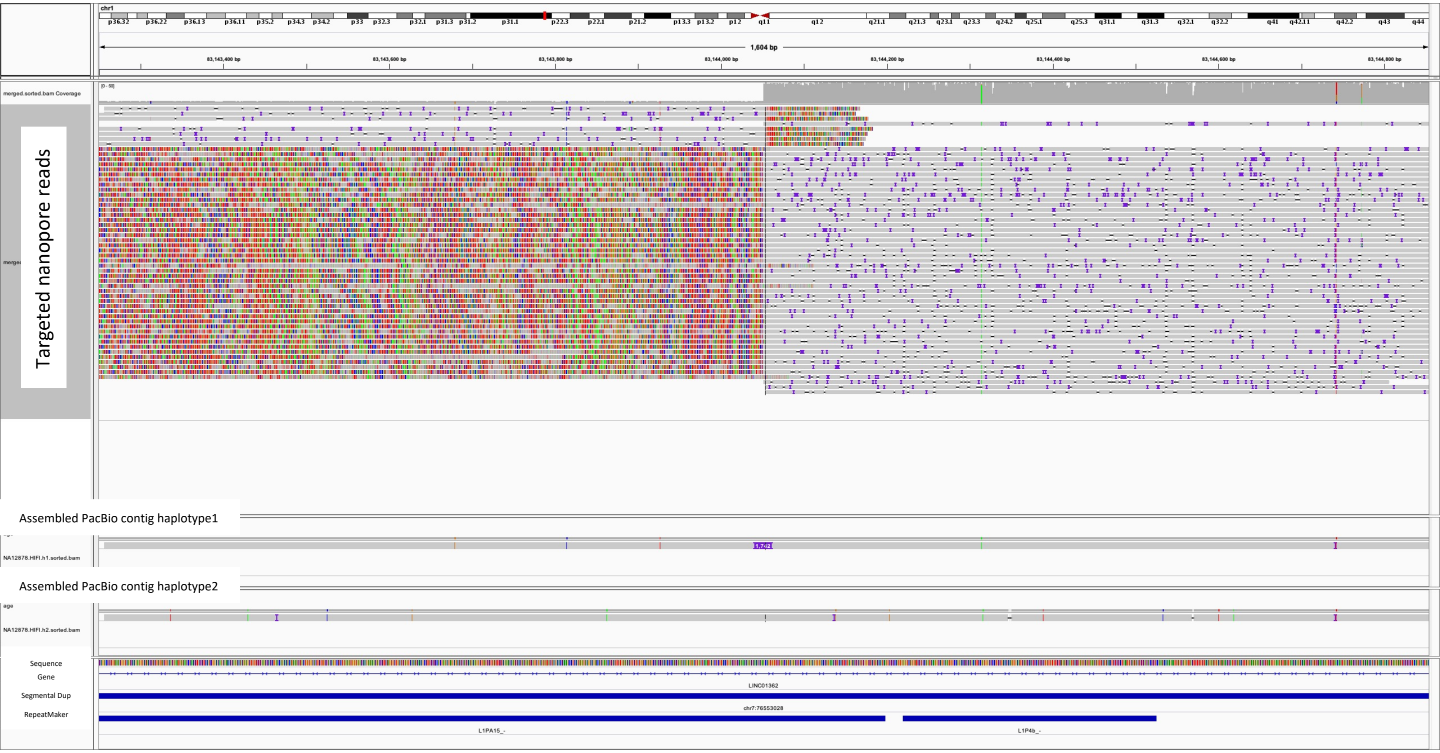

L1Hs call at chr2: 91993470

✧ General information

L1Hs insertion: 836bp L1Hs seq (-) 0/1

5′ GTCAGTTTATT AGTTTTCT Insertion AGTTTTCT AAAATAAAAAAA 3′

➢ Empty site

5′ GTCAGTTTATT AGTTTTCT AAAATAAAAAAA 3′
3′ CAGTCAAATAA TCAAAAGA TTTTATTTTTTT 5′

➢ Hallmarks

TSD length: 8bp
polyA length: 35bp
EN Cleavage site: 5′ TTCT/A 3′

➢ In Ebert et al. 2021. Science

NA12878 genotype: 0/0
Sample frequency: 7/32
Allele frequency: 7/64

✧ IGV screenshot (general information can be defined by nanopore reads)

➢ True positive type1 (as missed by other technologies)

chr2:91,992,688-91,994,252

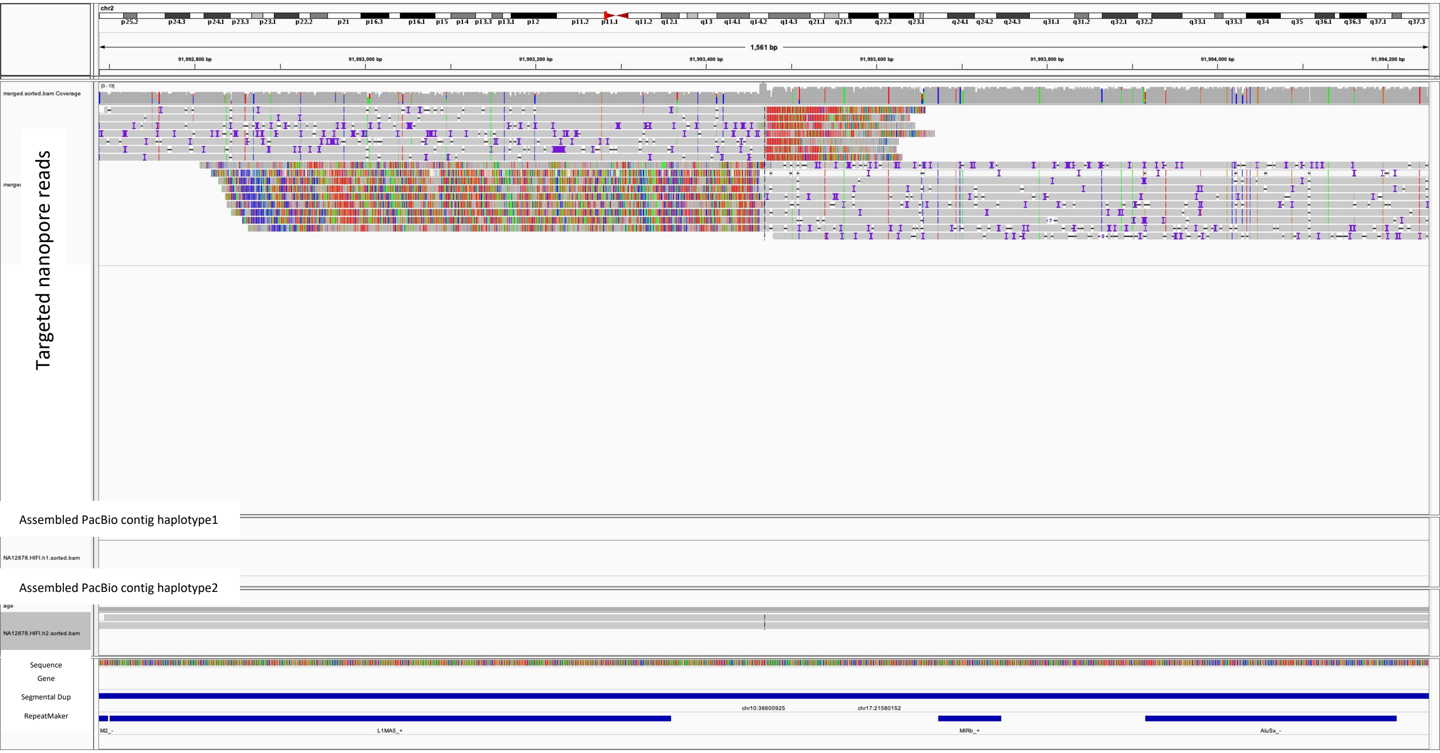

L1Hs call at chr2: 130095311

✧ General information

L1Hs insertion: full-length L1Hs seq (-) 0/1

5′ GTCAAACATTT CCATGTCAAACATTT **Insertion** CCATGTCAAACATTT TCAAAGTGAG 3′

➢ Empty site

5′ GTCAAACATTT CCATGTCAAACATTT TCAAAGTGAG 3′
3′ CAGTTTGTAAA GGTACAGTTTGTAAA AGTTTCACTC 5′

➢ Hallmarks

TSD length: 15bp
polyA length: 24bp
EN Cleavage site: 5′ ATTT/T 3′

➢ In Ebert et al. 2021. Science

NA12878 genotype:	0/0
Sample frequency:	4/32
Allele frequency:	4/64

✧ IGV screenshot (general information can be defined by nanopore reads)

➢ True positive type1 (as missed by other technologies)

chr2:130,089,051-130,101,572

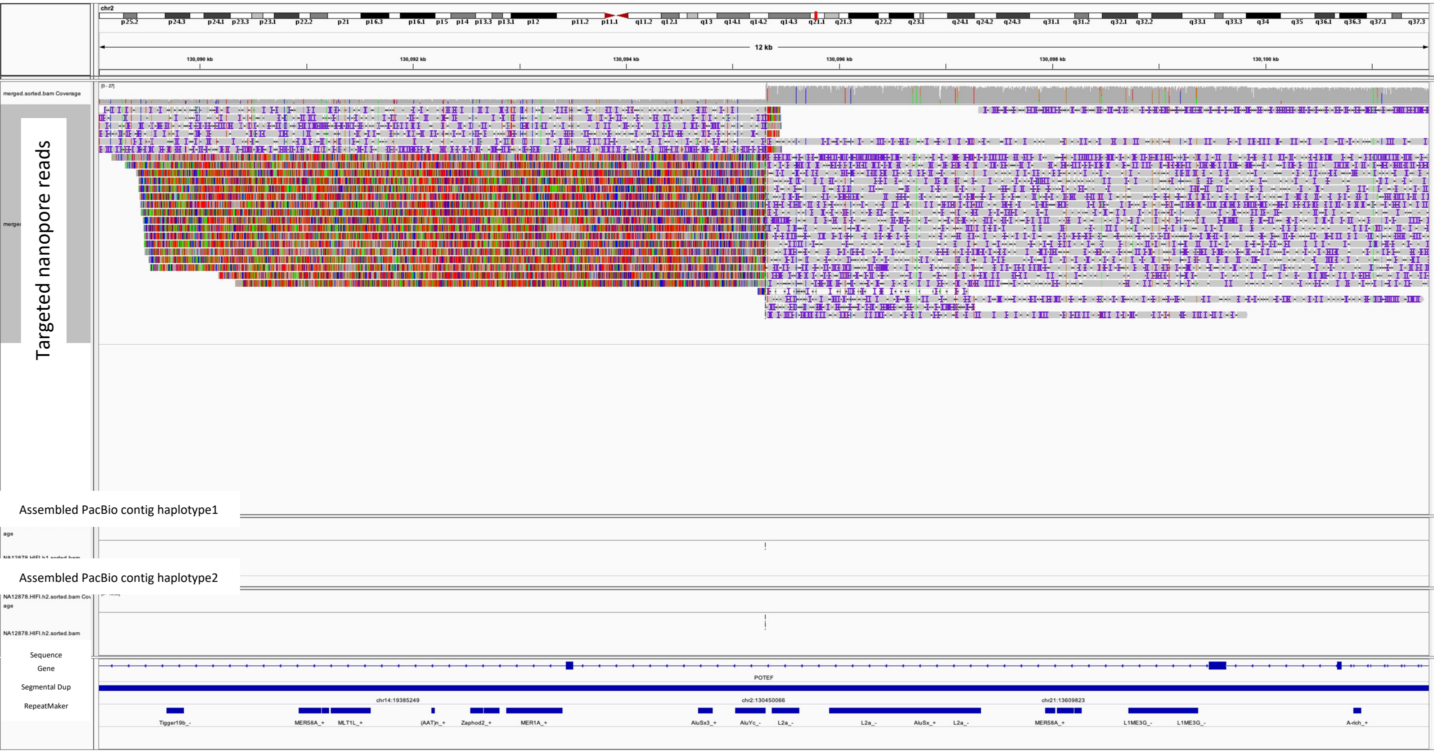

L1Hs call at chr2: 155671310

✧ General information

L1Hs insertion: full-length L1Hs seq (+) 0/1

5' AAACTCTGT CAAAAAAAAAAAAAGAAAGAAAGAAAGAA   CAAAAAAAAAAAAAGAAAGAAAGAAAGAA AGAAATAAAA 3'

Insertion

➢ Empty site

5' AAACTCTGT CAAAAAAAAAAAAAGAAAGAAAGAAAGAA AGAAATAAAA 3'
3' TTTGAGACA GTTTTTTTTTTTTTCTTTCTTTCTTTCTT TCTTTATTTT 5'

➢ Hallmarks

TSD length: 28bp
polyA length: 9bp
EN Cleavage site: 5' TTTG/A 3'

➢ In Ebert et al. 2021. Science

NA12878 genotype:	0/1 (mis-categorized)
Sample frequency:	12/32
Allele frequency:	16/64

✧ IGV screenshot (general information can be defined by nanopore reads)

➢ True positive type2 (as missed or mis-categorized by other callers, PALMER and PAV)

chr2:155,665,049-155,677,570

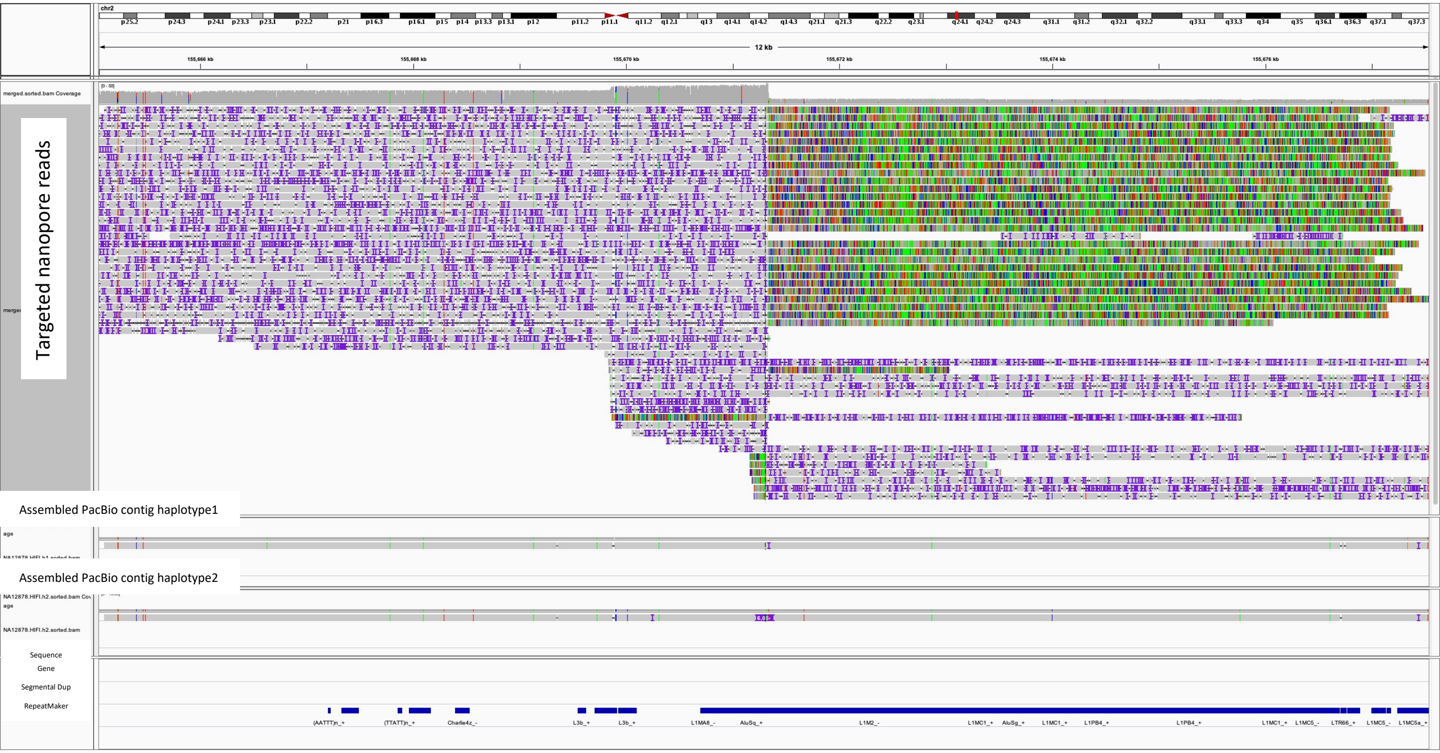

AluY call at chr2: 242181271

✧ General information

L1Hs insertion: 190bp AluY seq (+) 0/1

5′ TTCTCTTG AGATGCCCCAATC **Insertion** AGATGCCCCAATC ATCATT 3′

➢ Empty site

5′ TTCTCTTG AGATGCCCCAATC ATCATT 3′
3′ AAGAGAAC TCTACGGGGTTAG TAGTAA 5′

➢ Hallmarks

TSD length: 13bp
polyA length: 39bp
EN Cleavage site: 5' ATCT/C 3'

➢ In Ebert et al. 2021. Science

NA12878 genotype: 0/0
Sample frequency: 14/32
Allele frequency: 14/64

✧ IGV screenshot (general information can not be defined by nanopore reads)

➢ True positive type2 (as missed by other callers, PALMER and PAV)

chr2:242,180,491-242,182,055

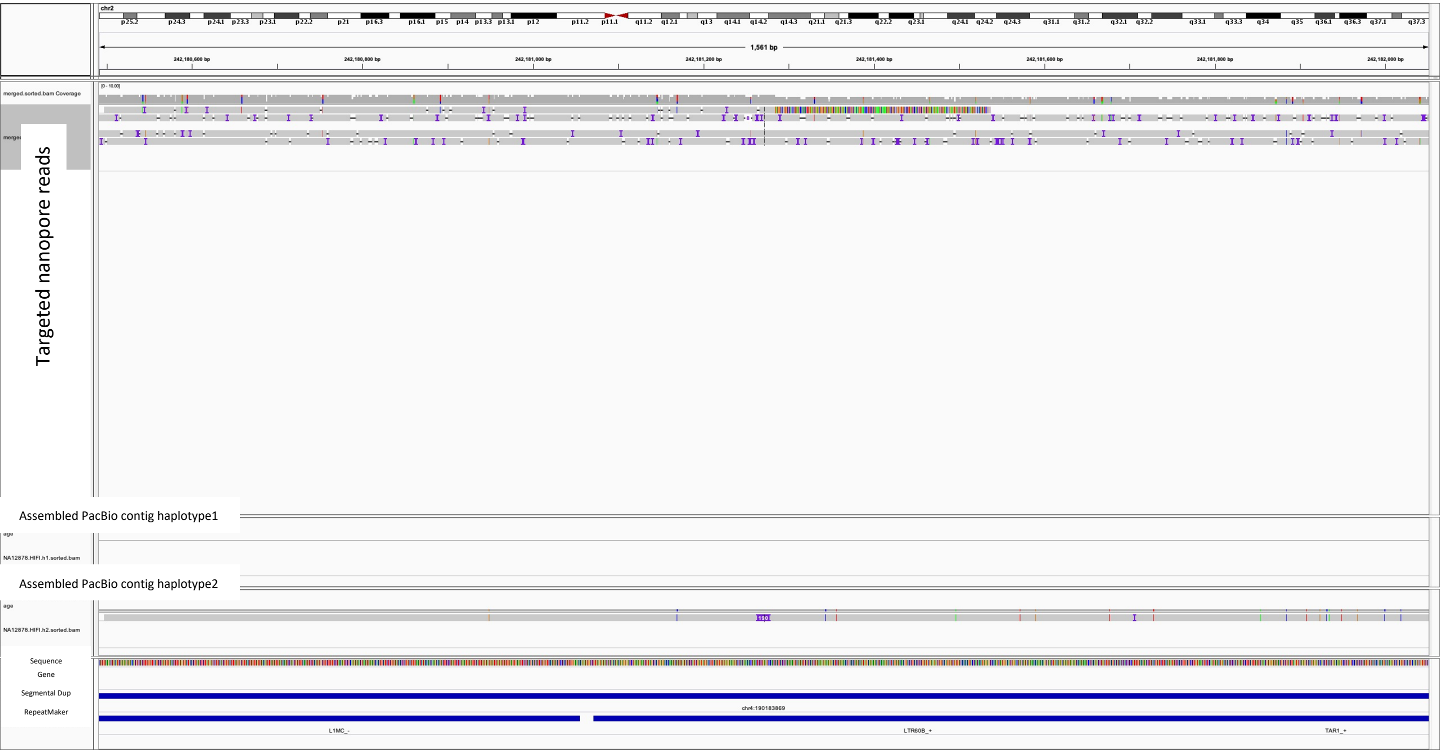

AluY call at chr5: 17712479

✧ General information

L1Hs insertion: 190bp AluY seq (+) 0/1

5′ TTTATCTCTT AGAAAATATACA **Insertion** AGAAAAATATACA CAAGAAAT 3′

➢ Empty site

5′ TTTATCTCTT AGAAAAATATACA CAAGAAAT 3′
3′ AAATAGAGAA TCTTTTTATATGT GTTCTTTA 5′

➢ Hallmarks

TSD length: 13bp
polyA length: 22bp
EN Cleavage site: 5′ TTCT/A 3′

➢ In Ebert et al. 2021. Science

NA12878 genotype: 0/0
Sample frequency: 28/32
Allele frequency: 39/64

✧ IGV screenshot (general information can be defined by nanopore reads)

➢ True positive type2 (as missed by other callers, PALMER and PAV)

chr5:17,711,913-17,713,085

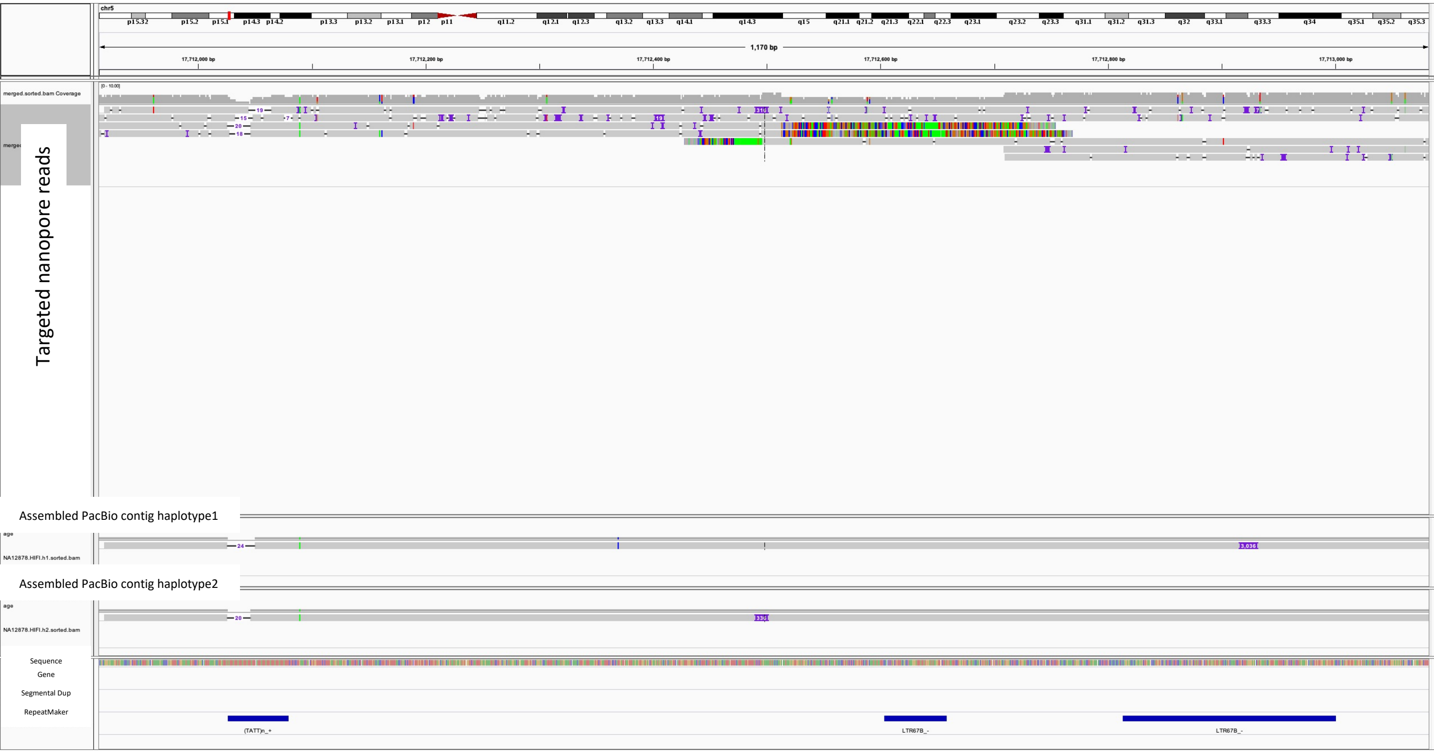

L1Hs call at chr6: 16950602

✧ General information

L1Hs insertion: 736bp L1Hs seq (-) 0/1

5′ CAATATCTGGA <u>AAATAG</u> **Insertion** <u>AAATGG</u> TATTTCTA 3′

➢ Empty site

5′ CAATATCTGGA AAATAG TATTTCTA 3′
3′ GTTATAGACCT TTTATC ATAAAGAT 5′

➢ Hallmarks

TSD length: 6bp
polyA length: 21bp
EN Cleavage site: 5’ ATAG/T 3’

➢ In Ebert et al. 2021. Science

NA12878 genotype:	0/1
Sample frequency:	5/32
Allele frequency:	7/64

✧ IGV screenshot (general information can be defined by nanopore reads)

➢ True positive type2 (as missed by other callers, PALMER and PAV)

chr6:16,950,051-16,951,153

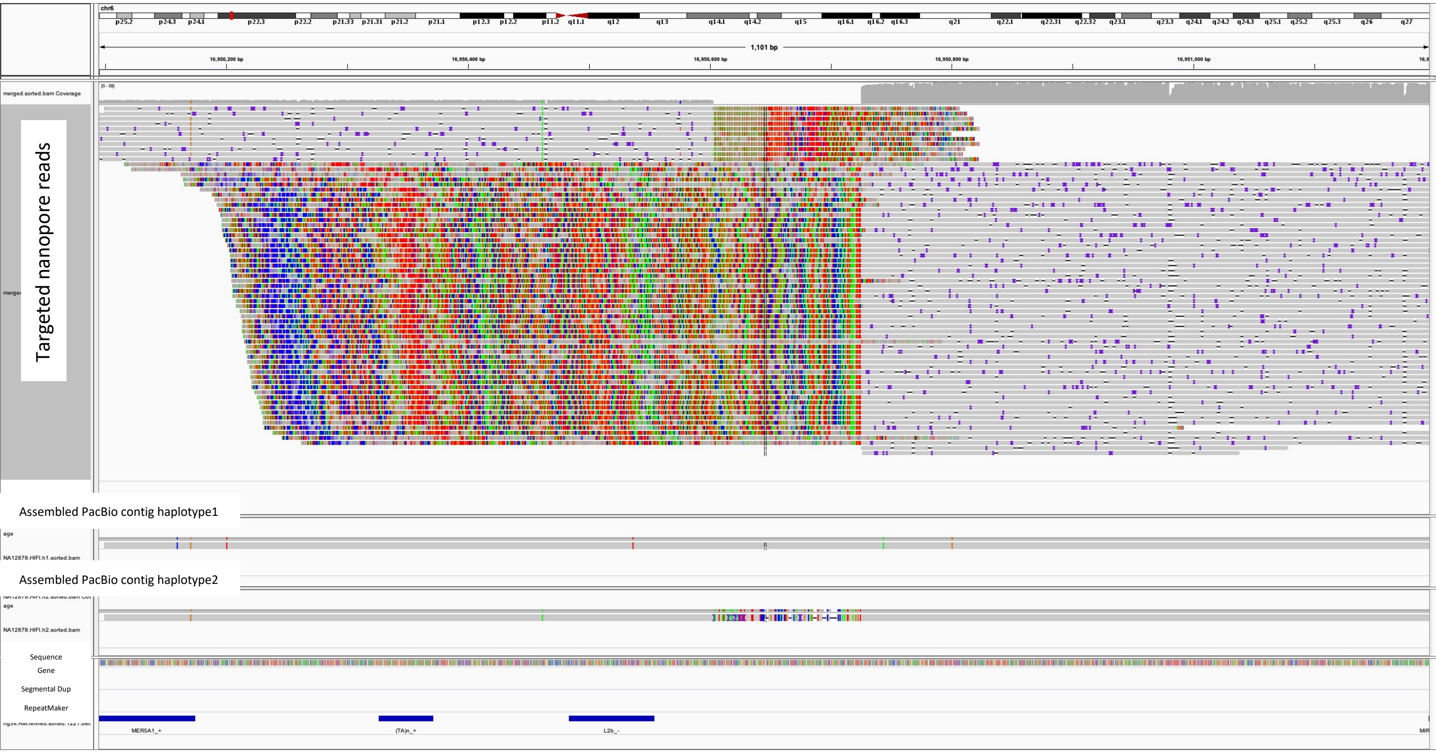

L1Hs call at chr6: 126302449

✧ General information

L1Hs insertion: 389bp L1Hs seq (-) 0/1

5’ ACTGACTGCTTT TGTT Insertion TGTT AATTGTCTCTC 3’

➢ Empty site

5’ ACTGACTGCTTT TGTT AATTGTCTCTC 3’
3’ TGACTGACGAAA ACAA TTAACAGAGAG 5’

➢ Hallmarks

TSD length: 4bp
polyA length: 74bp
EN Cleavage site: 5’ TGTT/A 3’

➢ In Ebert et al. 2021. Science

NA12878 genotype: 0/0
Sample frequency: 2/32
Allele frequency: 2/64

✧ IGV screenshot (general information can be defined by nanopore reads)

➢ True positive type2 (as missed by other callers, PALMER and PAV)

chr6:126,302,174-126,302,724

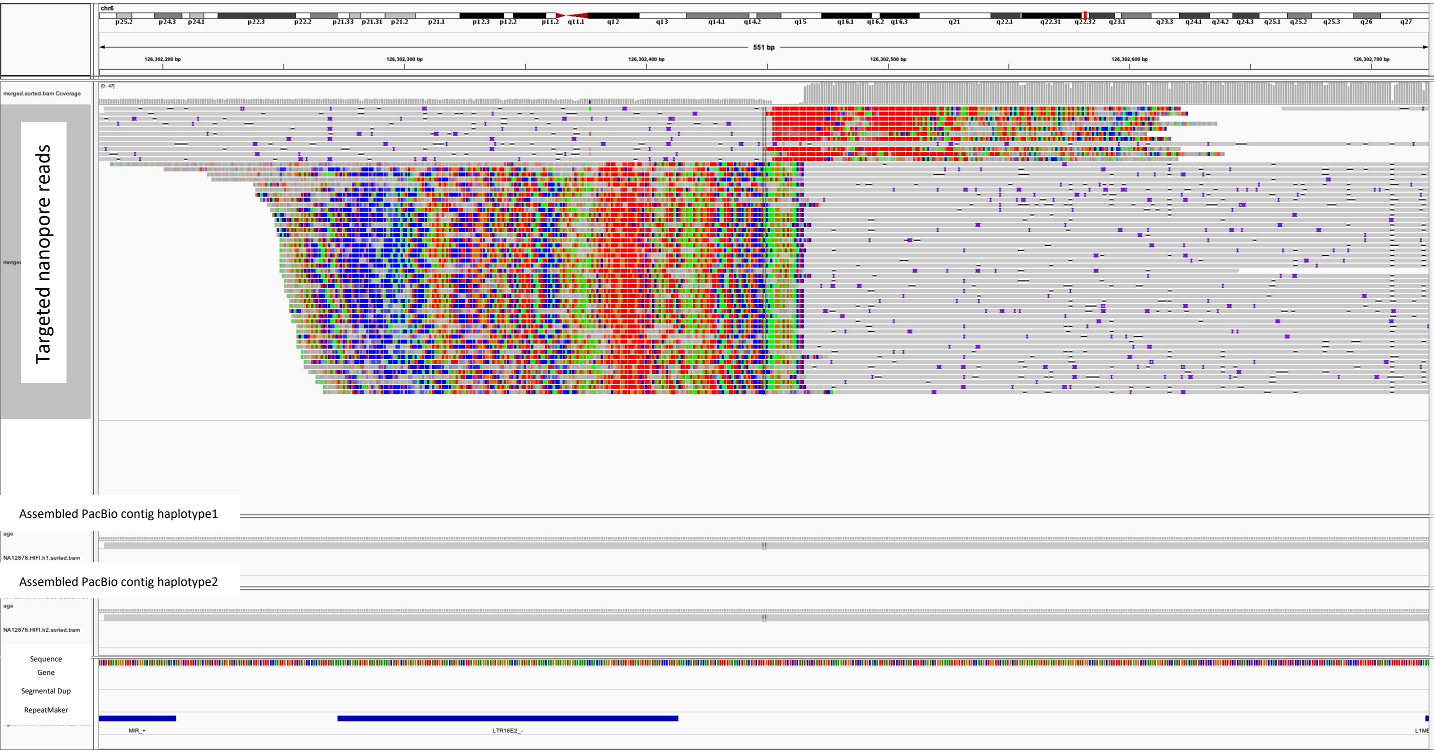

L1Hs call at chr10: 4997072

✧ General information

L1Hs insertion: 420bp L1Hs seq (-) 0/1

5′ TGACCACTGT TAGCTTAACCAA **Insertion** TAGCTTAACCAA GTTTTTAAA 3′

➢ Empty site

5′ TGACCACTGT TAGCTTAACCAA GTTTTTAAA 3′
3′ ACTGGTGACA ATCGAATTGGTT CAAAAATTT 5′

➢ Hallmarks

TSD length: 12bp
polyA length: 14bp
EN Cleavage site: 5’ CCAA/G 3’

➢ In Ebert et al. 2021. Science

NA12878 genotype: 0/0
Sample frequency: 4/32
Allele frequency: 5/64

✧ IGV screenshot (general information can not be defined by nanopore reads)

➢ True positive type1 (as missed by other technologies)

chr10:4,996,640-4,997,503

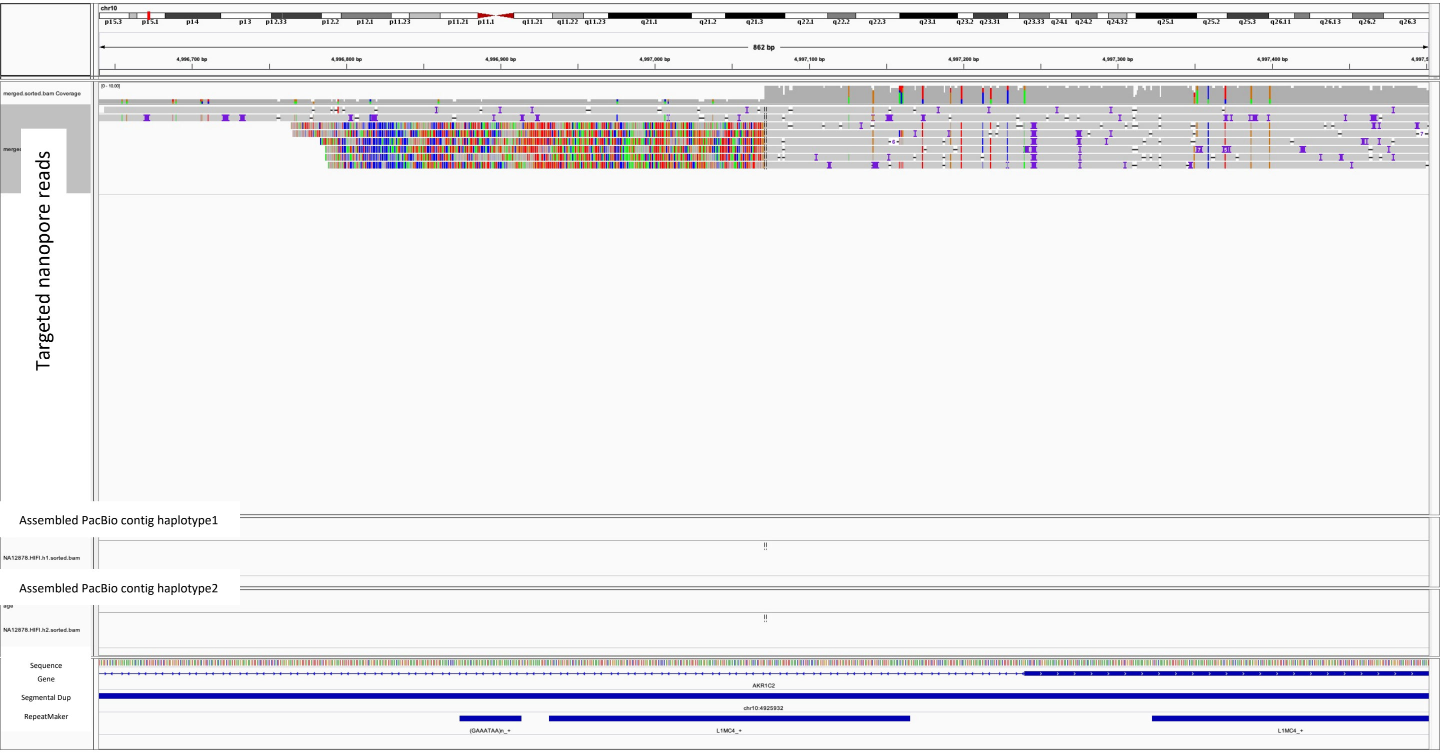

L1Hs call at chr10: 38748664

✧ General information

L1Hs insertion: 823bp L1Hs seq (-) 0/1

5′ TCAGTTTATT <u>AGTTTTCT</u> **Insertion** <u>AGTTTTCT</u> AAAATAAAAAAAA 3′

➢ Empty site

5′ TCAGTTTATT AGTTTTCT AAAATAAAAAAAA 3′
3′ AGTCAAATAA TCAAAAGA TTTTATTTTTTTT 5′

➢ Hallmarks

TSD length: 8bp
polyA length: 75bp
EN Cleavage site: 5′ TTCT/A 3′

➢ In Ebert et al. 2021. Science

NA12878 genotype: 0/0
Sample frequency: 0/32
Allele frequency: 0/64

✧ IGV screenshot (general information can be defined by nanopore reads)

➢ True positive type2 (as missed by other callers, PALMER and PAV)

chr10:38,747,800-38,749,528

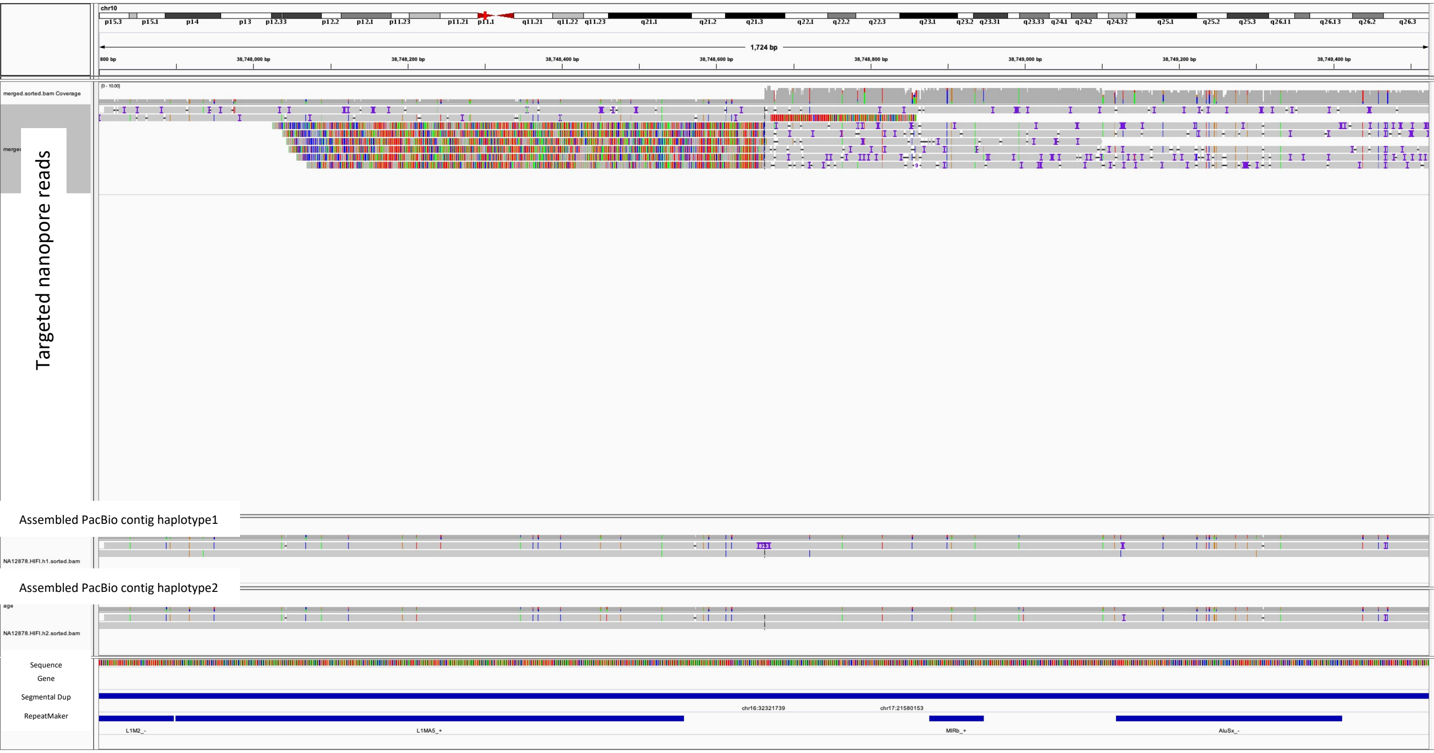

AluY call at chr16: 69157891

✧ General information

L1Hs insertion: 323bp AluY seq (-) 0/1

5’ CTGTGCCTGGC CCAAAAGTATTC **Insertion** CCAAAAGTATTC TTATACAT 3’

➢ Empty site

5’ CTGTGCCTGGC CCAAAAGTATTC TTATACAT 3’
3’ TACACGGACCG GGTTTTCATAAG AATATGTA 5’

➢ Hallmarks

TSD length: 12bp
polyA length: 26bp
EN Cleavage site: 5’ ATTC/T 3’

➢ In Ebert et al. 2021. Science

NA12878 genotype: 0/0
Sample frequency: 9/32
Allele frequency: 10/64

✧ IGV screenshot (general information can be defined by nanopore reads)

➢ True positive type2 (as missed by other callers, PALMER and PAV)

chr16:69,157,321-69,158,487

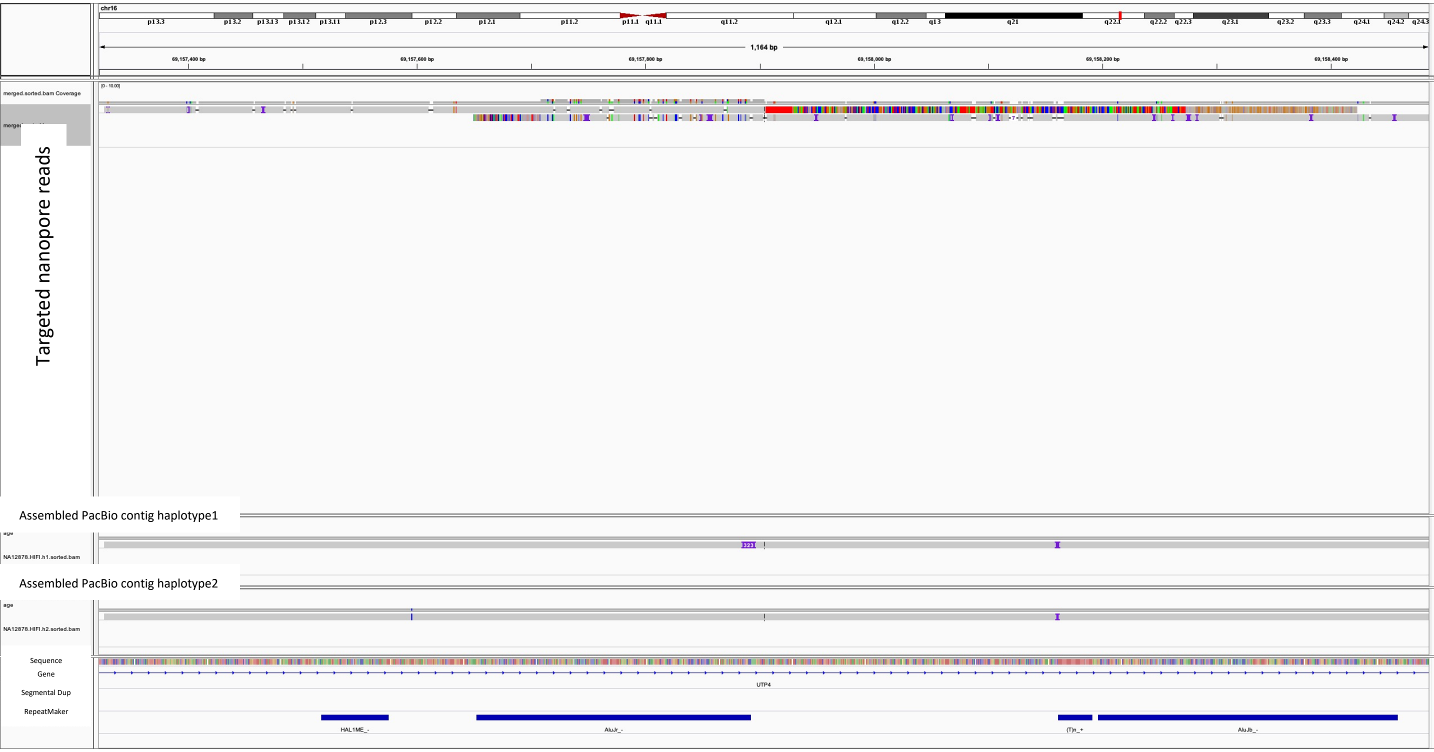

L1Hs call at chr17: 18755862

✧ General information

L1Hs insertion: 1202bp L1Hs seq (-) 0/1

5' TTAAGAACCT TGACCCAACATTTTCT **Insertion** TGACCCAACATTTTCT AACTCATGG 3'

➢ Empty site

5' TTAAGAACCT TGACCCAACATTTTCT AACTCATGG 3'
3' AATTCTTGGA ACTGGGTTGTAAAAGA TTGAGTACC 5'

➢ Hallmarks

TSD length: 16bp
polyA length: 20bp
EN Cleavage site: 5' TTCT/A 3'

➢ In Ebert et al. 2021. Science

NA12878 genotype:	0/0
Sample frequency:	1/32
Allele frequency:	1/64

✧ IGV screenshot (general information can be defined by nanopore reads)

➢ True positive type1 (as missed by other technologies)

chr17:18,754,586-18,757,137

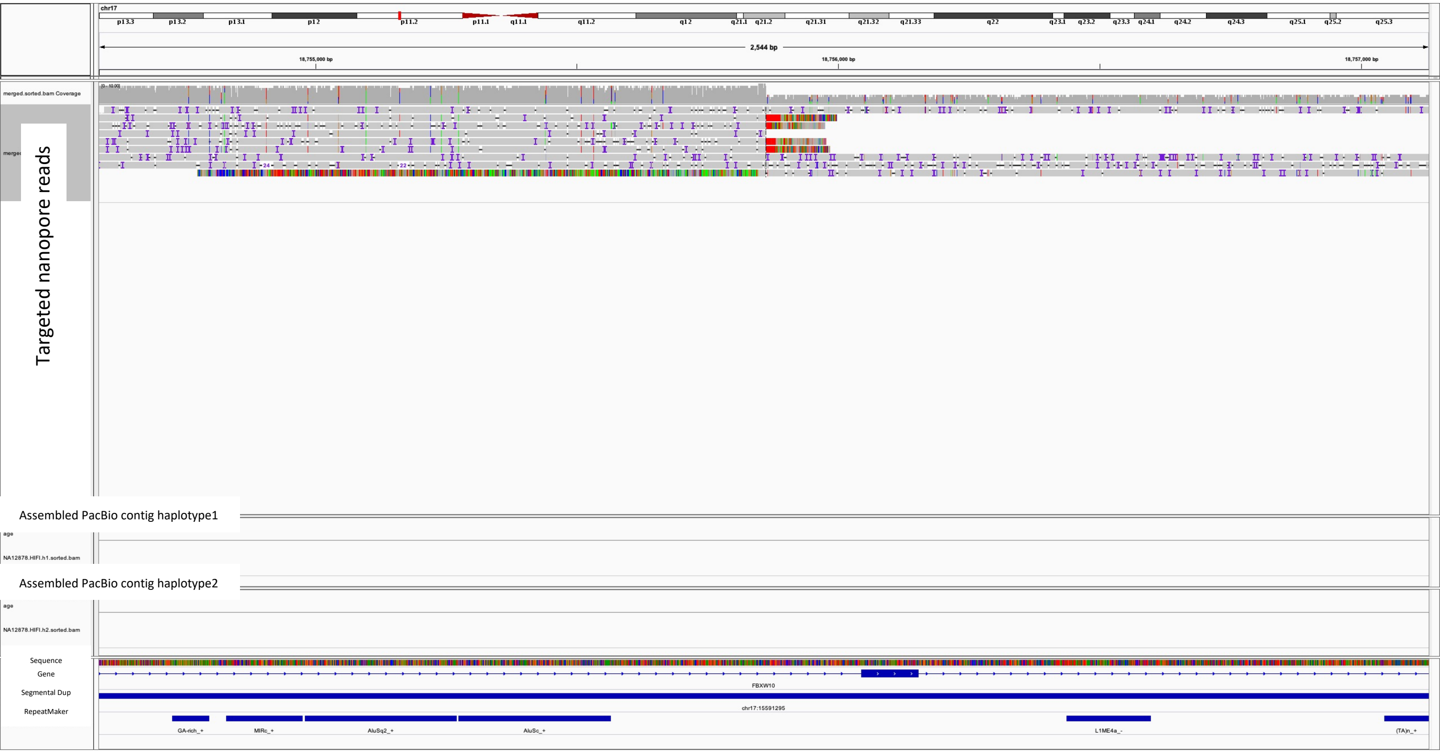

L1Hs call at chr17: 21828248

✧ General information

L1Hs insertion: 1300bp L1Hs seq (+) 0/1 (with a 700bp 5' inverted L1 segment)

5′ TTAATTCTATT GAAAAA **Insertion** GAAAAA CCAAATCTGTAGGAA 3′

➢ Empty site

5′ TTAATTCTATT GAAAAA CCAAATCTGTAGGAA 3′
3′ AATTAAGATAA CTTTTT GGTTTAGACATCCTT 5′

➢ Hallmarks

TSD length: 6bp
polyA length: 29bp
EN Cleavage site: 5' TTCT/A 3'

➢ In Ebert et al. 2021. Science

NA12878 genotype: 0/0
Sample frequency: 1/32
Allele frequency: 1/64

✧ IGV screenshot (general information can not be defined by nanopore reads)

➢ True positive type2 (as missed by other callers, PALMER and PAV)

chr17:21,827,696-21,828,771

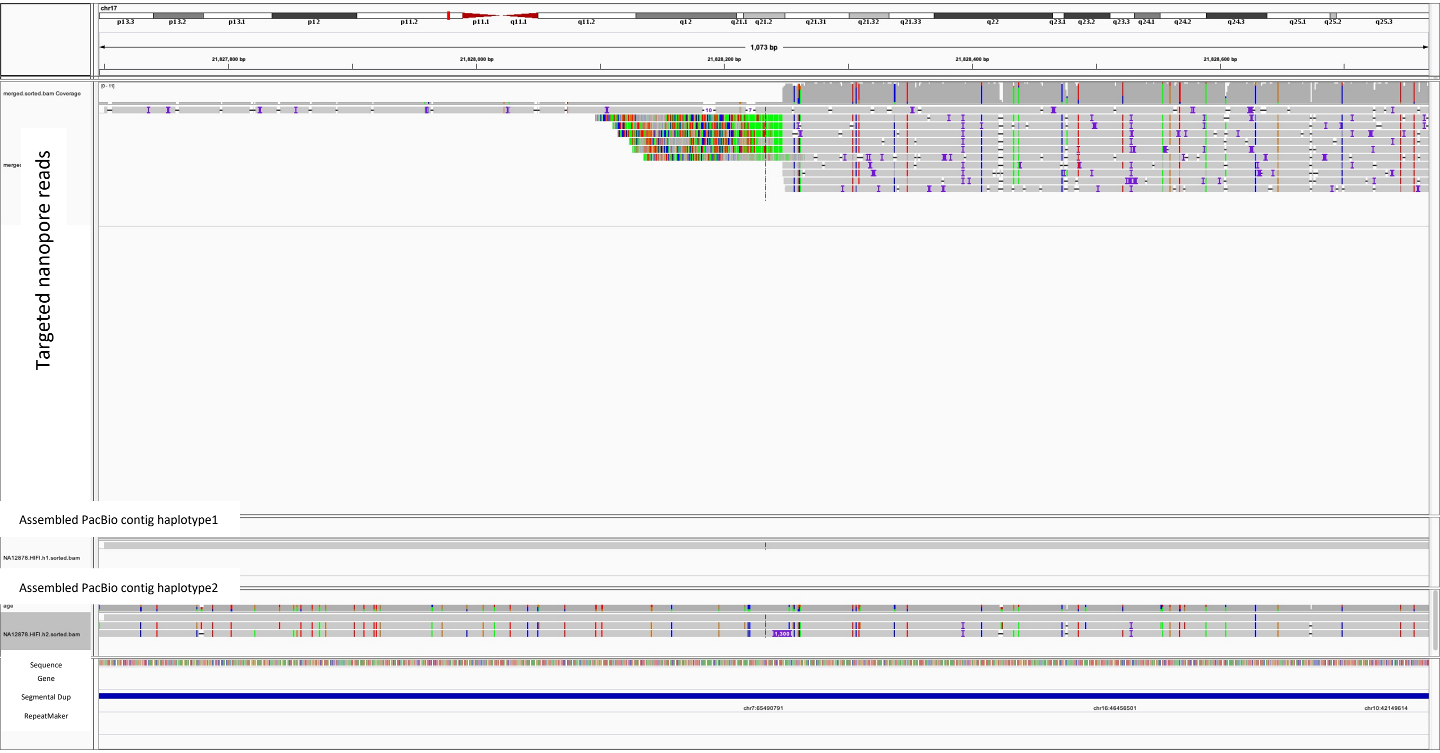

AluY call at chr19: 52384798

✧ General information

L1Hs insertion: 467bp AluY seq (+) 1/1 (with a 158bp 3' end segment)

5' ACGGGAGAGCA ACCTTACAAATGTAATGAATTTGG Insertion ACCTTACAAATGTAATGAATGTGG CAAAGCAT 3'

➢ Empty site

5' ACGGGAGAGCA ACCTTACAAATGTAATGAATGTGG CAAAGCAT 3'
3' TGCCCTCTCGT TGGAATGTTTACATTACTTACACC GTTTCGTA 5'

➢ Hallmarks

TSD length: 24bp
polyA length: 39bp
EN Cleavage site: 5' AGGT/T 3'

➢ In Ebert et al. 2021. Science

NA12878 genotype:	0/0
Sample frequency:	9/32
Allele frequency:	10/64

✧ IGV screenshot (general information can be defined by nanopore reads)

➢ True positive type2 (as missed by other callers, PALMER and PAV)

chr19:52,384,416-52,385,172

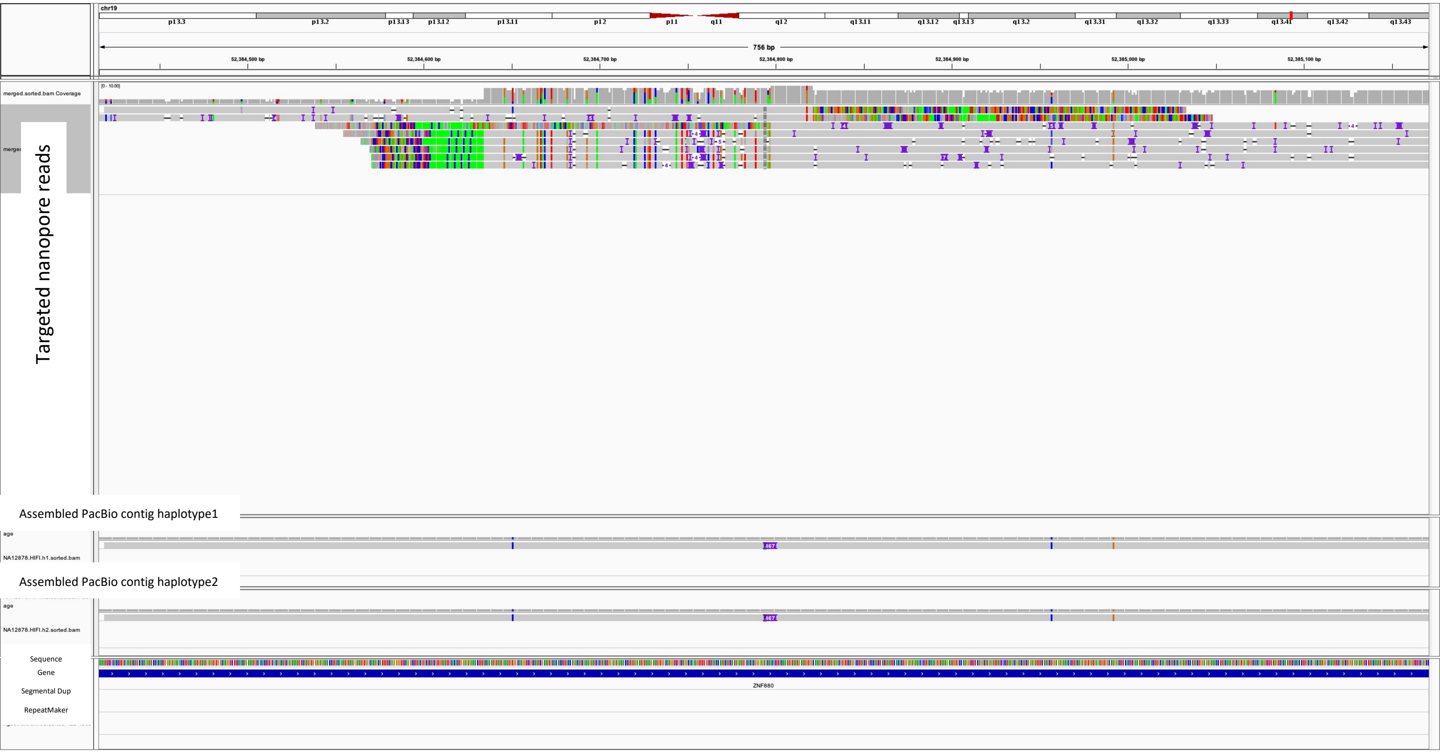

AluY call at chr19: 58605628

✧ General information

L1Hs insertion: 322bp AluY seq (+) 0/1

5′ GAACTTCTCT TAAGGTGCCCCAATC **Insertion** TGAGATGCCCCAATC ATCATTCACT 3′

➢ Empty site

5′ GAACTTCTCT TAAGGTGCCCCAATC ATCATTCACT 3′
3′ CTTGAAGAGA ATTCCACGGGGTTAG TAGTAAGTGA 5′

➢ Hallmarks

TSD length: 15bp
polyA length: 42bp
EN Cleavage site: 5′ CTTA/A 3′

➢ In Ebert et al. 2021. Science

NA12878 genotype:	0/0
Sample frequency:	10/32
Allele frequency:	13/64

✧ IGV screenshot (general information can be defined by nanopore reads)

➢ True positive type2 (as missed by other callers, PALMER and PAV)

chr19:58,605,246-58,606,002

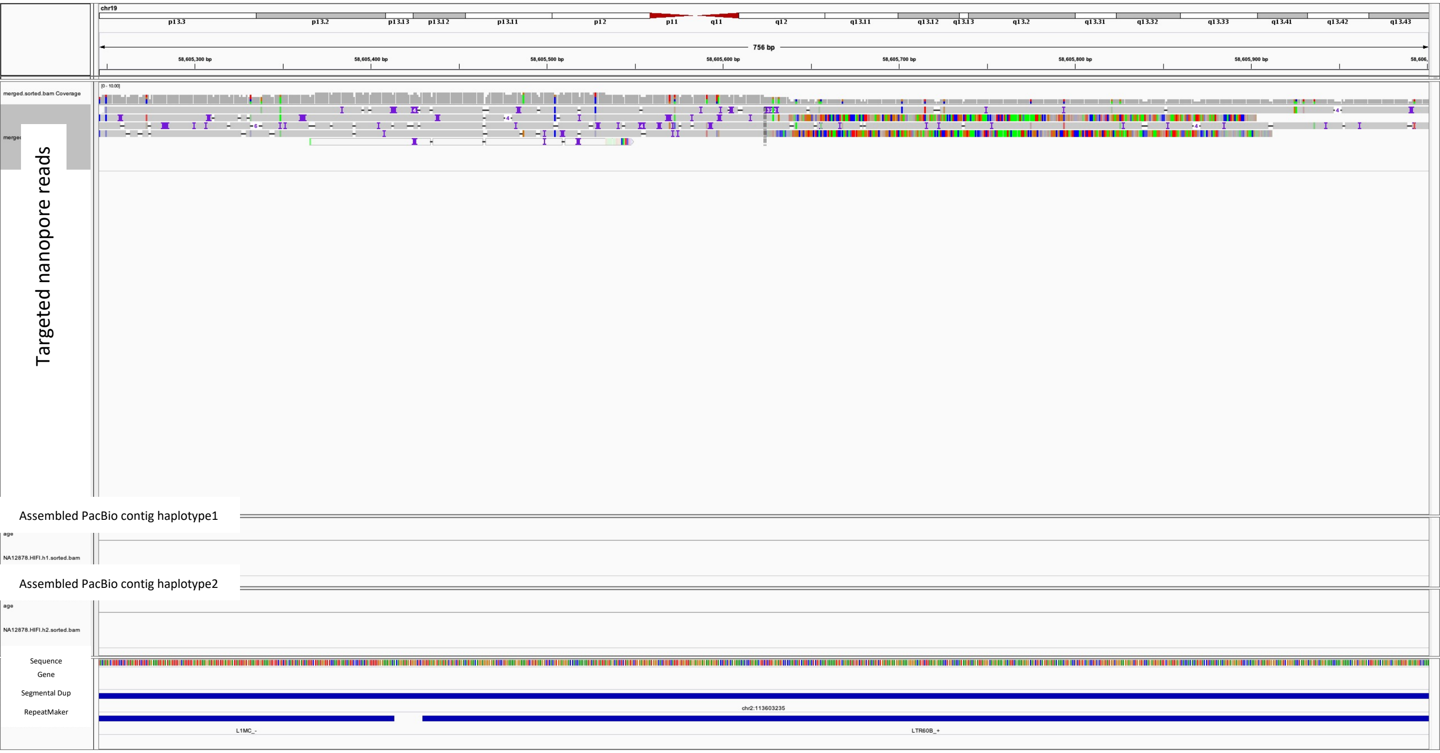

L1Hs call at chr20: 30176989

✧ General information

L1Hs insertion: 1011bp L1Hs seq (-) 0/1 (with 228bp 5' inverted L1 segment)

5′ tgtgttaggaa <u>aattccacatctttc</u> **Insertion** <u>aattccacatctttc</u> ctctagccatt 3′

➢ Empty site

5′ tgtgttaggaa aattccacatctttc ctctagccatt 3′
3′ acacaatcctt ttaaggtgtagaaag gagatcggtaa 5′

➢ Hallmarks

TSD length: 15bp
polyA length: 16bp
EN Cleavage site: 5' TTTC/C 3'

➢ In Ebert et al. 2021. Science

NA12878 genotype: 0/0
Sample frequency: 0/32
Allele frequency: 0/64

✧ IGV screenshot (general information can be defined by nanopore reads)

➢ True positive type1 (as missed by other technologies)

chr20:30,175,323-30,178,654

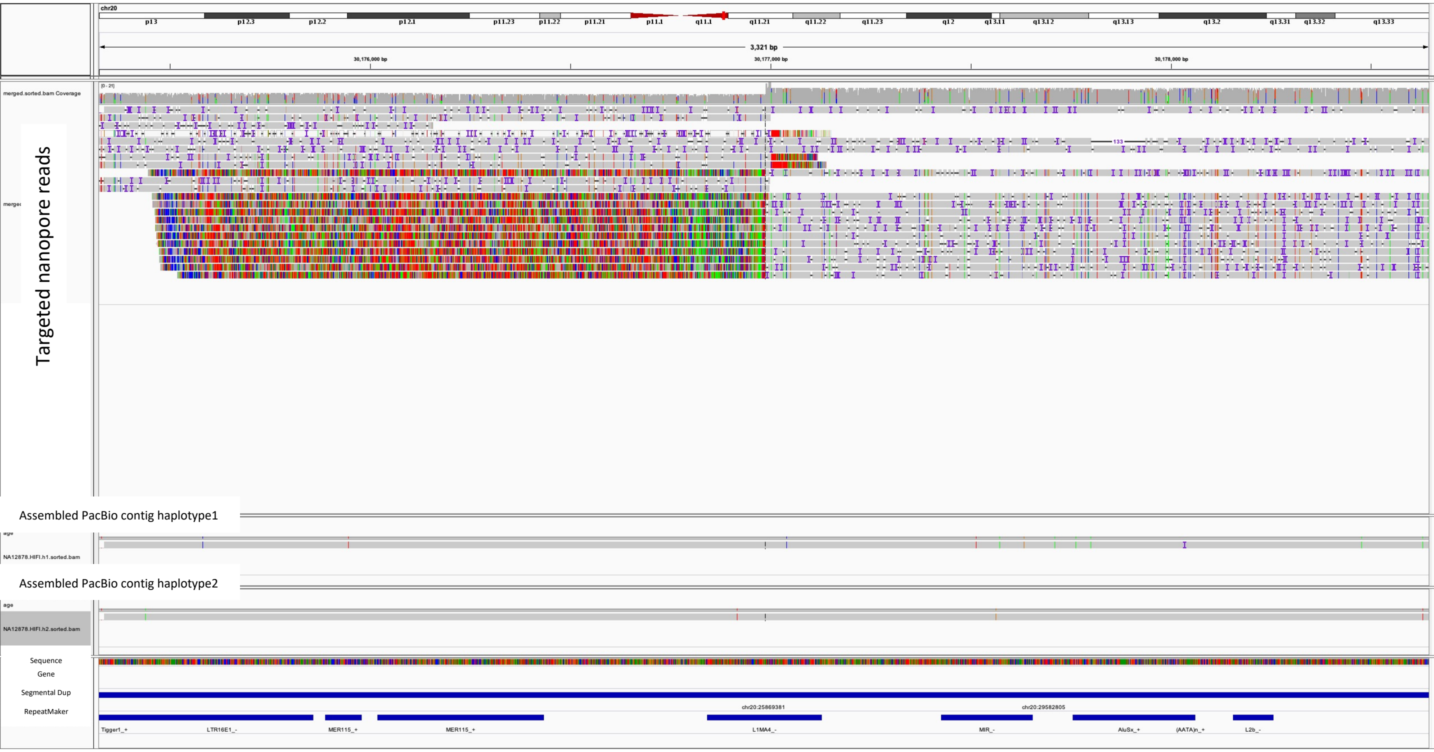

L1Hs call at chrX: 121709076

✧ General information

L1Hs insertion: 403bp L1Hs seq (-) 0/1

5′ GACTGGGT TTTTCTTT **Insertion** TTTTCTTT TACTGGTA 3′

➢ Empty site

5′ GACTGGGT TTTTCTTT TACTGGTA 3′
3′ CTGACCCA AAAAGAAA ATGACCAT 5′

➢ Hallmarks

TSD length: 7bp
polyA length: 75bp
EN Cleavage site: 5’ CTTT/A 3’

➢ In Ebert et al. 2021. Science

NA12878 genotype: 0/0
Sample frequency: 0/32
Allele frequency: 0/64

✧ IGV screenshot (general information can be defined by nanopore reads)

➢ True positive type1 (as missed by other technologies)

chrX:121,708,565-121,709,572

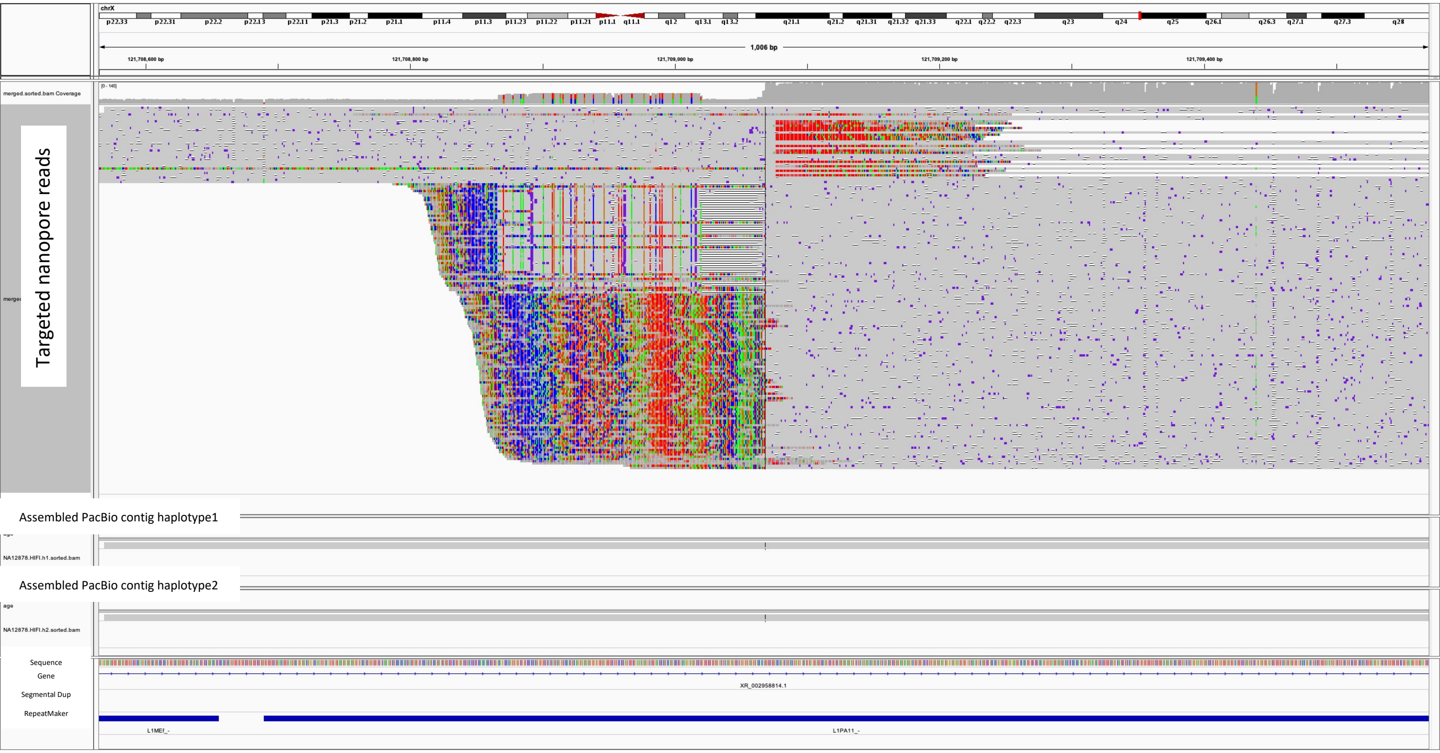

Supplement: Supplementary file 11 — Supplementary Dataset 9 [file 41467_2021_23918_MOESM11_ESM.pdf]
