# Supplementary material for: Cas9 targeted enrichment of mobile elements using nanopore sequencing
Source: Nat Commun. 2021 Jun 11;12:3586. doi: 10.1038/s41467-021-23918-y (PMC8196195; doi:10.1038/s41467-021-23918-y)
Supplement: Supplementary file 13 — Reporting Summary [file 41467_2021_23918_MOESM13_ESM.pdf]

nature research

Corresponding author(s): Alan P Boyle
Ryan E Mills

Last updated by author(s): May 14, 2021

Reporting Summary

Nature Research wishes to improve the reproducibility of the work that we publish. This form provides structure for consistency and transparency in reporting. For further information on Nature Research policies, see our Editorial Policies and the Editorial Policy Checklist.

Statistics

For all statistical analyses, confirm that the following items are present in the figure legend, table legend, main text, or Methods section.

n/a	Confirmed	
☐	☒	The exact sample size (*n*) for each experimental group/condition, given as a discrete number and unit of measurement
☒	☐	A statement on whether measurements were taken from distinct samples or whether the same sample was measured repeatedly
☐	☒	The statistical test(s) used AND whether they are one- or two-sided<br>*Only common tests should be described solely by name; describe more complex techniques in the Methods section.*
☒	☐	A description of all covariates tested
☒	☐	A description of any assumptions or corrections, such as tests of normality and adjustment for multiple comparisons
☐	☒	A full description of the statistical parameters including central tendency (e.g. means) or other basic estimates (e.g. regression coefficient) AND variation (e.g. standard deviation) or associated estimates of uncertainty (e.g. confidence intervals)
☐	☒	For null hypothesis testing, the test statistic (e.g. *F*, *t*, *r*) with confidence intervals, effect sizes, degrees of freedom and *P* value noted<br>*Give P values as exact values whenever suitable.*
☒	☐	For Bayesian analysis, information on the choice of priors and Markov chain Monte Carlo settings
☒	☐	For hierarchical and complex designs, identification of the appropriate level for tests and full reporting of outcomes
☒	☐	Estimates of effect sizes (e.g. Cohen's *d*, Pearson's *r*), indicating how they were calculated

Our web collection on statistics for biologists contains articles on many of the points above.

Software and code

Policy information about availability of computer code

Data collection	MinKNOW/20.06.5<br>samtools/1.3.1 https://github.com/samtools/samtools<br>ncbi-blast++/2.10.0 ftp://ftp.ncbi.nlm.nih.gov/blast/executables/blast+/LATEST/<br>PALMER https://github.com/mills-lab/PALMER/releases/tag/V1.7.2<br>minimap2 https://github.com/lh3/minimap2<br>jellyfish/2.2.8<br>nanopolish/0.13.2 https://github.com/jts/nanopolish<br>methylartist https://github.com/adamewing/methylartist<br>Porechop/0.2.4 https://github.com/rrwick/Porechop<br>Guppy/4.0.15 Oxford Nanopore Technologies
Data analysis	All script and pipelines in this publication, including Nano-Pal, are available on GitHub: https://github.com/Boyle-Lab/NanoPal-and-Cas9-targeted-enrichement-pipelines.<br>The enhanced version of PALMER is available at https://github.com/mills-lab/PALMER.

For manuscripts utilizing custom algorithms or software that are central to the research but not yet described in published literature, software must be made available to editors and reviewers. We strongly encourage code deposition in a community repository (e.g. GitHub). See the Nature Research guidelines for submitting code & software for further information.

Data

Policy information about availability of data

All manuscripts must include a data availability statement. This statement should provide the following information, where applicable:

- Accession codes, unique identifiers, or web links for publicly available datasets
- A list of figures that have associated raw data
- A description of any restrictions on data availability

The nanopore sequencing data for the Cas9 targeted enrichment of MEIs in this study are available in the SRA repository under BioProject accession PRJNA699027. GRCh38 (GRCh38+decoy) reference genome obtained from the 1000 Genomes Project (ftp://ftp.1000genomes.ebi.ac.uk/vol1/ftp/technical/reference/GRCh38_reference_genome/)
https://www.ncbi.nlm.nih.gov/sra/PRJNA699027

Field-specific reporting

Please select the one below that is the best fit for your research. If you are not sure, read the appropriate sections before making your selection.

☒ Life sciences ☐ Behavioural & social sciences ☐ Ecological, evolutionary & environmental sciences

For a reference copy of the document with all sections, see nature.com/documents/nr-reporting-summary-flat.pdf

Life sciences study design

All studies must disclose on these points even when the disclosure is negative.

Sample size	Study was not preempted by power analysis. The only consideration of sample size would regard the number of reads and ratios of enrichment, which is an indeterminate prior performing the sequencing experiments.
Data exclusions	No data were excluded.
Replication	Replication of MEI enrichment was accomplished across individual flongles and pooled MinION experiments. Each subfamily of MEI in this study was successfully replicated for Cas9 targeted enrichment and nanopore sequencing at least twice.
Randomization	Randomization was not relevant to this study as there were no participants/biases/parameters of our enrichment and sequencing experiments to randomize in a meaningful way that would eliminate variability in the data or introduce rigorousness to the approach.
Blinding	Blinding was not necessary as there are no participants or biases to blind from the experiments that might otherwise influence the results.

Reporting for specific materials, systems and methods

We require information from authors about some types of materials, experimental systems and methods used in many studies. Here, indicate whether each material, system or method listed is relevant to your study. If you are not sure if a list item applies to your research, read the appropriate section before selecting a response.

Materials & experimental systems

n/a	Involved in the study	
☒	☐	Antibodies
☐	☒	Eukaryotic cell lines
☒	☐	Palaeontology and archaeology
☒	☐	Animals and other organisms
☒	☐	Human research participants
☒	☐	Clinical data
☒	☐	Dual use research of concern

Methods

n/a	Involved in the study	
☒	☐	ChIP-seq
☒	☐	Flow cytometry
☒	☐	MRI-based neuroimaging

Eukaryotic cell lines

Policy information about cell lines

Cell line source(s)	GM12878, GM12891, and GM12892 were acquired from Coriell Biobank.
Authentication	GM12878, GM12891, and GM12892 were provided with a certificate of authentication from Coriell. Species of origin was confirmed by Nucleoside Phosphorylase, Glucose-6-Phosphate Dehydrogenase, and Lactate Dehydrogenase Isoenzyme Electrophoresis. Family relationships have been verified by Southern blot hybridization with minisatellite (VNTR) probes or by PCR using a panel of microsatellite markers. Gender has been verified by PCR with a Y chromosome-specific primer pair. Replicate cultures or matched cultures of differing cell types from the same individual have been analyzed by PCR using microsatellite and Y chromosome-specific primer pairs to assure cell culture identity.<br>We additionally authenticated cell lines by matching expected variation from the individuals to publicly available data.
Mycoplasma contamination	All cell lines tested negative for mycoplasma contamination.
Commonly misidentified lines<br>(See ICLAC register)	No commonly misidentified cell lines were used in this study.
